# Bone marrow stromal cells dictate lanosterol biosynthesis and ferroptosis of multiple myeloma

**DOI:** 10.1038/s41388-024-03020-5

**Published:** 2024-04-09

**Authors:** Hongmei Jiang, Lijuan Wang, Qiguo Zhang, Sheng Wang, Linchuang Jia, Hao Cheng, Jingya Wang, Xin Li, Ying Xie, Yixuan Wang, Meilin Hu, Jing Guo, Qian Li, Ziyi Peng, Mengqi Wang, Yangyang Xie, Tiantian Li, Yafei Wang, Bill D. Geng, Sundararaman Swaminathan, P. Leif Bergsagel, Zhiqiang Liu

**Affiliations:** 1 Department of Pathology, Sichuan Provincial People’s Hospital, University of Electronic Science and Technology of China, Chengdu, Sichuan 610072 China; 2 Central Laboratory, Linyi People’s Hospital, Linyi, Shandong Province 276037 China; 3 Department of Hematology, The First People’s Hospital of Chuzhou, Chuzhou Hospital Affiliated to Anhui Medical University, Chuzhou, 239000 China; 4 Department of Hematology, Nanjing Drum Tower Hospital, Nanjing University, Nanjing, Jiangsu 210008 China; 5 The Province and Ministry Co-Sponsored Collaborative Innovation Center for Medical Epigenetics; Tianjin Key Laboratory of Cellular Homeostasis and Human Diseases, School of Basic Medical Science; Department of Physiology and Pathophysiology, School of Basic Medical Science, Tianjin Medical University, Heping, Tianjin, 300070 China; 6 School of Stomatology, Tianjin Medical University, Heping, Tianjin, 300070 China; 7 Tianjin Medical University Cancer Institute and Hospital; National Clinical Research Center for Cancer; Tianjin Key Laboratory of Cancer Prevention and Therapy; Tianjin’s Clinical Research Center for Cancer, Tianjin, 300192 China; 8 School of Natual Science, University of Texas at Austin, Austin, TX 78712 USA; 9 Division of Nephrology and Hypertension, Mayo Clinic Arizona, Scottsdale, AZ 85259 USA; 10 Division of Hematology/Oncology, Mayo Clinic Arizona, Scottsdale, AZ 85259 USA; 11 The Proton Center of Shandong Cancer Institute and Hospital, Shandong First Medical University and Shandong Academy of Medical Science, Jinan, Shandong 250117 China

**Keywords:** Cell death, Myeloma

## Abstract

Ferroptosis has been demonstrated a promising way to counteract chemoresistance of multiple myeloma (MM), however, roles and mechanism of bone marrow stromal cells (BMSCs) in regulating ferroptosis of MM cells remain elusive. Here, we uncovered that MM cells were more susceptible to ferroptotic induction under the interaction of BMSCs using in vitro and in vivo models. Mechanistically, BMSCs elevated the iron level in MM cells, thereby activating the steroid biosynthesis pathway, especially the production of lanosterol, a major source of reactive oxygen species (ROS) in MM cells. We discovered that direct coupling of CD40 ligand and CD40 receptor constituted the key signaling pathway governing lanosterol biosynthesis, and disruption of CD40/CD40L interaction using an anti-CD40 neutralizing antibody or conditional depletion of Cd40l in BMSCs successfully eliminated the iron level and lanosterol production of MM cells localized in the Vk*MYC Vk12653 or NSG mouse models. Our study deciphers the mechanism of BMSCs dictating ferroptosis of MM cells and highlights the therapeutic potential of non-apoptosis strategies for managing refractory or relapsed MM patients.

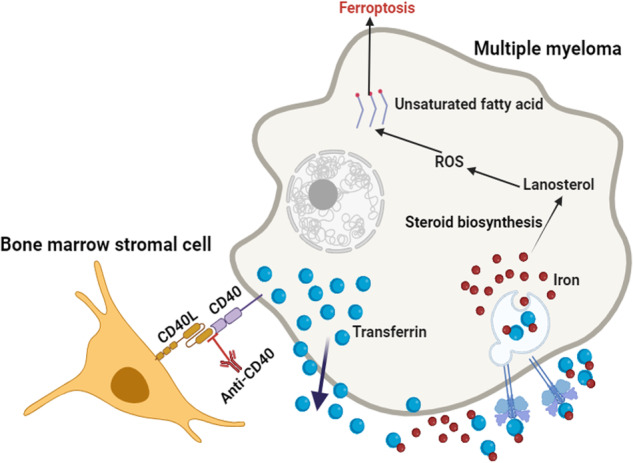

## Introduction

Given the formidable challenge posed by chemo-resistance in multiple myeloma (MM) therapy, there is an imperative to explore innovative therapeutic approaches targeting MM. Ferroptosis, characterized by iron-dependent programmed cell death and the accumulation of reactive oxygen species (ROS) [1], emerges as a promising avenue. Key triggers for ferroptosis include disorders or dysfunctions of iron metabolism, system X_c_^−^, GPX4 protein, and lipid peroxidation [2]. A growing body of literature suggests ferroptosis as a key target in cancer treatments [3]. Hangauer *et al* discovered that the GPX4 inhibitor RSL3, effectively reversed drug resistance in solid tumor [4], while the system X_c_^−^ inhibitor erastin, demonstrated the ability to increase the sensitivity of acute myeloid leukemia to chemotherapies [5]. However, the exploration of ferroptosis in MM remains limited. MM patients commonly experience anemia due to dysregulated iron metabolism, as expanding malignant MM cells deprive the uptake of iron required for erythron development [6], thereby laying the foundation for ferroptosis in MM cells. Nevertheless, mechanisms of ferroptosis in MM progression, especially its viability as a target for managing refractory or relapsed (RR) MM patients, remain largely unexplored.

Bone marrow environment, comprising of bone marrow stromal cells (BMSCs) and non-cell components, is recognized as an essential for the survival and progression of MM, an incurable plasma cell malignancy, facilitated through the secretion of cytokines or direct cellular contact [7–9]. Moreover, BMSCs, capable of differentiating into adipocytes, contribute to increased chemoresistance in MM cells and exacerbate bone lesions in the patients [10, 11]. Interactions between MM cells and BMSCs, mediated by α4β1/VCAM-1, MUC1/ICAM-1, and CD40/CD40L, promote the initiation and progression of MM [12]. While various therapeutic interventions targeting bone marrow microenvironment, such as an IL-6 targeting monoclonal antibody siltuximab [13, 14], have been applied, however, the outcomes of these clinical trials remain largely unsatisfactory, indicating that only a partial interference with the interaction of MM and BMSC has been achieved. Therefore, an in-depth investigation into the mechanism governing the interactome of MM and BMSCs is imperative for the development of effective therapeutic strategies.

In addition, some researchers have proposed that tumor environment exhibits either anti-tumor or pro-tumor effects by influencing the promotion or suppression of ferroptosis of cancer cells. CD8+ T cells within the tumor environment can stimulate ferroptosis in tumor cells through the release of IFNγ [15], whereas lymphocytes protect metastasizing melanoma cells from ferroptosis [16]. Various metabolites within the tumor environment can also alter the sensitivity of tumor cells to ferroptosis; for instance, lactate may promote the resistance of liver cells to RSL3 [17], while n-3 and n-6 polyunsaturated fatty acids could lead to ferroptosis-mediated anticancer effects [18]. Thus, investigating whether BMSCs, acting as the tumor environment of MM, promote or suppress ferroptosis of MM cells is a topic worthy of exploration.

In the current study, we aim to elucidate the role and mechanism of BMSCs in regulating iron metabolism, decipher their role in shifting iron surplus to cholesterol and lanosterol, and explore how BMSCs counteract ferroptosis in MM cells. Our results reveal that BMSCs sensitize MM cells to ferroptosis-based therapy by generating ROS from lanosterol through the activation of steroid biosynthesis pathway. These novel findings advance our understanding of the MM interactome within the bone marrow niche, offering therapeutic potential for developing new strategies for RRMM in the clinic.

## Results

### BMSCs shift MM cells response from resistance to ferroptosis-based therapy

**Fig. 1:**
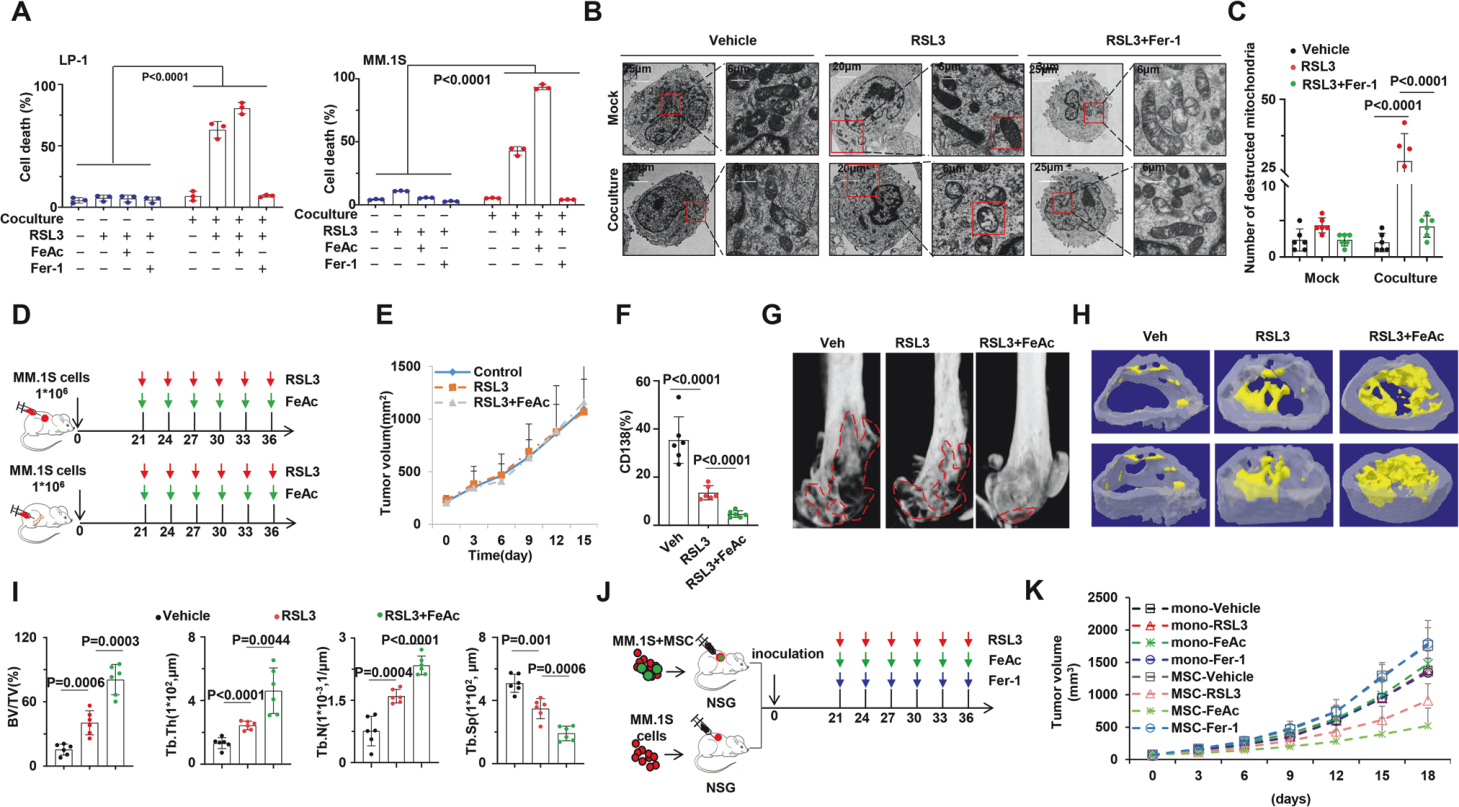
BMSCs sensitize MM cells to RSL3. **A** Statistical analysis of flow cytometry assay showing ferroptosis level determined by PI in mock and co-cultured MM cells induced by RSL3 (*n* = 3). LP-1, RSL3 (1 μM), Fer-1 (100 μM), FeAc (100 μM). MM.1S, RSL3 (0.5 μM), Fer-1 (100 μM), FeAc (100 μM). **B** Transmission electron microscopy (TEM) analysis of mock and co-cultured MM.1 S cells, illustrating ferroptosis level induced by RSL3, and **C** corresponding statistical analysis showing number of destructed mitochondria in MM.1S cells. Mock indicates MM.1S cells only, and Co-culture indicates MM.1S co-cultured with BMSCs. **D** Schematic diagram illustrating the treatment of xenograft and intra-bone model derived from NSG mouse (*n* = 8). **E** Tumor growth of MM.1 S cells in xenograft model in mice treated with RSL3 with or without FeAc. Tumor volume = 1/2(L*W^2^) mm^3^, where the L presents the length and W represents the width of the tumor. **F** CD138 positive cells infiltration in bone marrow of mouse femurs bearing MM.1S cells. **G**, **H** Representative micro-CT reconstructions and 3D reconstructions of bone trabecula in metaphyseal region of mouse femurs bearing MM.1S cells. **I** Measurement of the percentage of bone volume to total volume (BV/TV), cortical thickness, number of bone trabecula, and trabecula separation in the metaphyseal region of the mice femur bearing MM.1S cells. **J** Schematic diagram illustrating the treatment of xenograft model using NSG mice by inoculation of a mixture of MM.1S-luci and BMSCs (*n* = 8). **K** Tumor growth of MM.1S cells alone or a mixture of MM.1S and BMSCs from xenograft model in NSG mouse treated with RSL3. *P* values are determined by unpaired two-sided t-tests with Welch’s correction.

Studies have demonstrated that tumor environment can influence drug-resistant solid tumor cells, rendering them more susceptible to ferroptosis inducers such as RSL3 and erastin [4, 17]. BMSCs, as a component of MM tumor environment, have been implicated in inducing multi-chemoresistance in MM cell [19, 20]. To elucidate whether MM cells exhibit similar responses, we investigated the impact of co-culturing MM cells with BMSCs. We observed that co-culture with BMSCs significantly enhanced the chemoresistance to BTZ in MM cells (Fig. s1A, s1B). Interestingly, MM cells from both the control and co-culture groups exhibited notable tolerance to erastin, a system Xc^−^ inhibitor, (Fig. s1C). However, co-cultured MM cells demonstrated increased sensitivity to RSL3, a GPX4 inhibitor. Administration of iron significantly augmented ferroptosis, while the lipid hydroperoxide inhibitor Ferrostatin-1 (Fer-1) markedly diminished ferroptosis in MM cells (Fig. 1A), under the condition where iron or Fer-1 alone couldn’t trigger MM cell death (Fig. s1D). The occurrence of ferroptosis in MM cells was further confirmed by the observed reduction or disappearance of mitochondria crista (Fig. 1B, C). Nevertheless, our previously established BTZ-resistant MM (BR-MM) cells (Fig. s1E) [21], cultured in the absence of BMSCs, did not exhibit increased sensitivity to RSL3 (Fig. s1F). To clarify the importance of BMSC in regulating MM ferroptosis in vivo, we established the xenograft and intra-bone MM growth models to mimic the control and co-cultured systems, respectively (Fig. 1D). Administration of RSL3 barely suppressed tumor growth in the xenograft model, irrespective of the presence of iron (Fig. 1E), but it notably eliminated CD138^+^ plasma cells in the bone marrow of the intra-bone model (Fig. 1F). In addition, significant remission of the bone lesion was observed in the RSL3 treatment group of the intra-bone model, especially when iron was supplemented (Fig. 1G–I). To exclude the influence of other BM stromal cells on MM cells in vivo, we constructed another xenograft model by inoculating MM cells with or without BMSCs under the skin of NSG mice (Fig. 1J). The results validated that RSL3 suppressed tumor growth only when BMSCs were present, and the role of RSL3 could be augment by FeAc while diminished by Fer-1 in the present of BMSCs (Fig. 1K). These findings strongly support the conclusion that BMSCs potentiate MM cells’ sensitivity to ferroptosis-based therapy.

### Iron levels are elevated in BMSCs interacting with MM cells

**Fig. 2:**
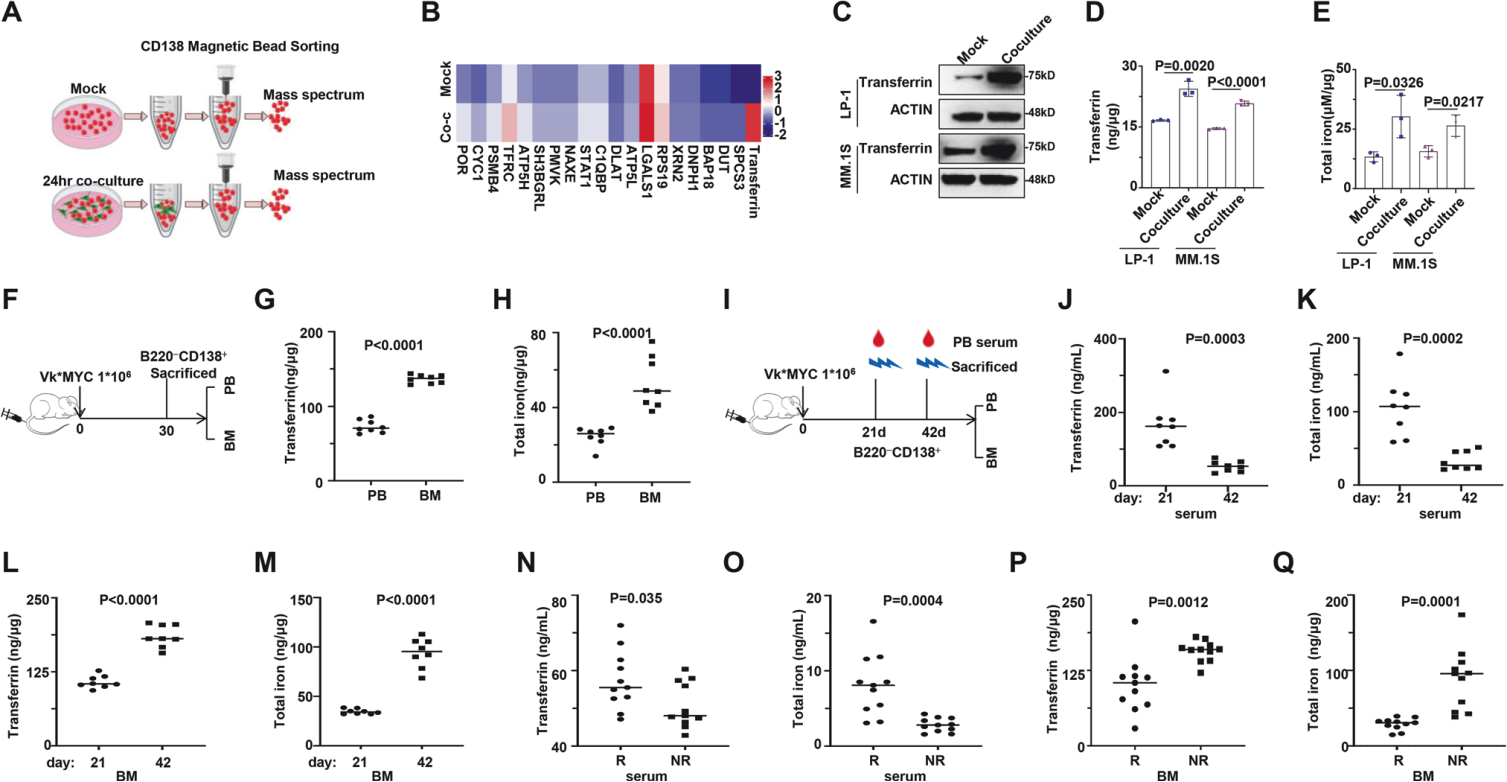
Iron metabolism is upregulated in co-culture treatment MM cells. **A** Schematic diagram illustrating the workflow of mock and co-cultured MM cell separation, where red indicates MM cells and green indicates BMSCs. The upper panel represents the Mock condition of MM.1S cells only; while the lower panel represents the Co-culture of MM.1S with BMSCs. **B** Heat map displaying top 20 upregulated proteins in whole cell lysates of mock and co-cultured MM.1S cells analyzed by mass spectrometry (MS) analysis. Red indicates higher expression and blue indicates lower expression. While mock indicates MM.1S cells only, Co-culture indicates MM.1 S co-cultured with BMSCs. **C** Representative western blotting showing transferrin levels in mock and co-cultured MM cells (*n* = 3). Mock indicates MM.1S cells only, and Co-culture indicates MM.1S co-cultured with BMSCs. **D** ELISA analysis of transferrin levels in mock and co-cultured MM cells (*n* = 3). Mock indicates MM.1 S cells only, and Co-culture indicates MM.1 S co-cultured with BMSCs. **E** Total iron levels in mock and co-cultured MM cells (*n* = 3). Mock indicates MM.1S cells only, and Co-culture indicates MM.1S co-cultured with BMSCs. **F** Schematic diagram illustrating the treatment of Vk*MYC Vk12653 mouse model (*n* = 8). **G** ELISA analysis showing transferrin levels in CD138^+^B220^−^ cells separated from peripheral blood and bone marrow of Vk*MYC Vk12653 mouse model (*n* = 8). **H** Total iron level in CD138^+^B220^-^ cells separated from peripheral blood and bone marrow of Vk*MYC Vk12653 mouse model of MM (*n* = 8). **I** Schematic diagram illustrating the treatment of Vk*MYC Vk12653 mouse model (*n* = 8). **J** ELISA analysis of transferrin levels in serum of peripheral blood derived from Vk*MYC Vk12653 mouse model of MM (*n* = 8). **K** Total iron levels in serum of peripheral blood derived from Vk*MYC Vk12653 mouse model of MM (*n* = 8). **L** ELISA analysis of transferrin levels in CD138^+^B220^−^ cells separated from bone marrow of Vk*MYC Vk12653 mouse model of MM (*n* = 8). **M** Total iron levels in CD138^+^B220^−^ cells separated from bone marrow of Vk*MYC Vk12653 mouse model of MM (*n* = 8). **N** ELISA analysis of transferrin levels in serum of responders (R) and non-responders (NR) MM patients. **O** Total iron levels in serum of responders (R) and non-responders (NR) MM patients. **P** ELISA analysis of transferrin levels in CD138 positive cells derived from responders (R) and non-responders (NR) MM patients. **Q** Total iron levels in CD138 positive cells derived from responders (R) and non-responders (NR) MM patients. P values are determined by unpaired two-sided t-tests with Welch’s correction.

To elucidate the mechanism by which BMSCs enhance the sensitivity of MM cells to RSL3, we performed a proteomics assay on BMSCs interacting MM.1S cells to identify the key regulators of BMSCs on MM cells (Fig. 2A). The analysis revealed a remarkable upregulation of transferrin, a glycoprotein that binds and delivers Fe^3+^ into cells through transferrin receptor, in co-cultured MM cells, along with the increased expression of transferrin receptor (TFRC) (Fig. 2B). Notably, the total transferrin protein levels (Fig. 2C), soluble transferrin (Fig. 2D), and total iron levels (Fig. 2E) in the co-cultured MM cells were all remarkably augmented. In vivo, we isolated CD138^+^B220^−^ cells from peripheral blood (PB) and bone marrow (BM) of the Vk*MYC Vk12653 mouse model of myeloma, closely mimicking the MM disease (Fig. 2F) [22]. The analysis revealed that BM-derived CD138^+^B220^-^ cells expressed significantly higher levels of transferrin and iron compared to those in PB-derived cells (Fig. 2G, H). Consistent with the cell toxicity results of RSL3 on MM cells, we observe no meaningful expression changes of transferrin at protein (Fig. s2A), mRNA (Fig. s2B), secreting levels (Fig. s2C), and total iron levels (Fig. s2D) between the BR-MM and WT-MM cells. Subsequently, we found that iron levels increased in MM cells with the progression of MM in the presence of BMSCs. In the Vk∗MYC Vk12653 tumor-bearing mice (Fig. 2I), transferrin and iron levels remarkably decreased in serum of peripheral blood (Fig. 2J, K), but both dramatically accumulated in the bone marrow-derived CD138^+^B220^−^ cells (Fig. 2L, M) from day 21 to day 42. Clinically, transferrin and iron levels in peripheral blood serum of MM patients were notably decreased in non-responders compared to responders to BTZ-based regimens (Fig. 2N, O). However, both transferrin and iron levels were markedly elevated in the CD138^+^ plasma cells isolated from BM (Fig. 2P, Q). Meanwhile, iron promoted chemoresistance of BTZ to MM cells, even when the role of iron in promoting the proliferation of MM cells was interrupted (Fig. s3A–E). Collectively, these findings suggest that the accumulation of iron in MM cells, dependent on BMSCs-formed tumor environment, amplifies with the progression of MM cells.

### Iron accumulation activates steroid biosynthesis pathway in BMSCs interacting with MM cells

**Fig. 3:**
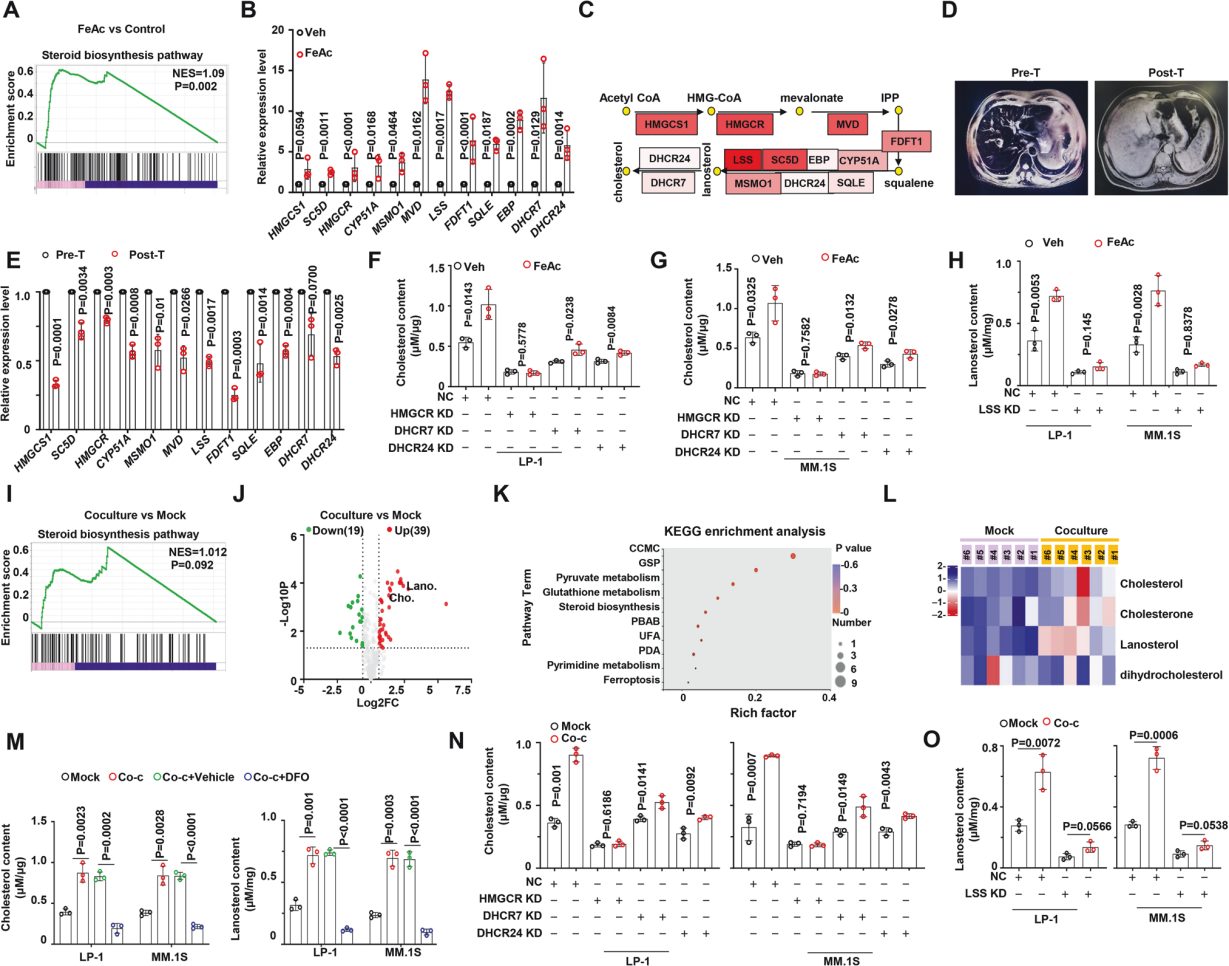
Interaction with BMSCs activates cholesterol biosynthesis pathway in MM cells by iron accumulation. **A** GSEA analysis reveals gene enrichment in steroid biosynthesis pathway in MM.1S cells treated with FeAc (300 μM) for 12 h. **B** Real-time PCR quantifying the mRNA level of genes involved in steroid biosynthesis pathway in vehicle and FeAc (300 μM) treatment in MM.1 S cells (*n* = 3). **C** Schematic diagram illustrating the process of steroid biosynthesis and expression of the pathway-related genes. **D** CT results showing the liver of an ALAS2 mutation patient. **E** Real-time PCR quantifying the mRNA level of genes involved in steroid biosynthesis pathway in B cells from the ALAS2 mutation patient. **F** Cholesterol content in NC and HMGCR-KD, DHCR7-KD, and DHCR24-KD LP-1 cells with or without FeAc (300 μM). NC none target control. **G** Cholesterol content in NC and HMGCR-KD, DHCR7-KD, and DHCR24-KD MM.1 S cells with or without FeAc (300 μM). NC, none target control. **H** Lanosterol content in NC and LSS-KD MM cells with or without FeAc (300 μM). NC none target control. **I** GSEA analysis showing gene enrichment in steroid biosynthesis pathway in mock and co-cultured MM.1S cells. **J** Volcano plot showing significantly differentially changed metabolites in control and co-cultured MM cells identified by Gas Chromatograph Mass Spectrometer (GC-MS). **K** KEGG analysis reveals the top 10 pathways enriched in co-cultured MM.1S cells. **L** Heat map shows metabolite abundance in steroid pathway in co-cultured MM.1S cells. Mock indicates MM.1S cells only, and Co-culture indicates MM.1S co-cultured with BMSCs. **M** Cholesterol and lanosterol content in mock and co-cultured MM cells with or without DFO (100 μM). Mock indicates MM cells only, and Co-culture indicates MM cells co-cultured with BMSCs. **N** Cholesterol content in NC and HMGCR-KD, DHCR7-KD, and DHCR24-KD MM.1S cells with or without coculture treatment. Mock indicates MM cells only, and Co-culture indicates MM cells co-cultured with BMSCs. NC, none target control. **O** Lanosterol content in NC and LSS-KD MM cells with or without co-culture treatment. Mock indicates MM cells only, and Co-culture indicates MM cells co-cultured with BMSCs. NC none target control. *P* values are determined by unpaired two-sided t-tests with Welch’s correction.

To further decipher the role of iron in MM cells, we conducted bulk RNA sequencing on MM.1S cells treated with iron. Differentially expressed genes (DEG) analysis showed 519 genes were downregulated, while 911 genes were upregulated significantly (Fig. s4A). Gene Ontology (GO) analysis demonstrated that the upregulated genes were enriched in cholesterol, sterol, and steroid metabolic processes (Fig. s4B). Kyoto Encyclopedia of Genes and Genomes (KEGG) analysis also indicated a significant enrichment of steroid biosynthesis pathway (Fig. s4C). Gene set enrichment analysis (GSEA) further suggested that iron promoted the activation of steroid biosynthesis pathway in MM cells (Fig. 3A). To discern the specific steps in the steroid biosynthesis pathway regulated by iron, we screened 12 key genes governing steroid biosynthesis in the RNA-seq data, and validated a remarkable upregulation in their expressions (Fig. 3B). These genes collectively participate in the entire pathway linking acetyl CoA to cholesterol (Fig. 3C). Importantly, plasma cells isolated from a patient with hereditary sideroblastic anemia (HAS) caused by ALAS2 mutation (1343G>A), which leads to failure of iron transportation and to iron overload in patient, (Fig. s4D) exhibited a partial restoration of gene expressions within the entire sterol biosynthesis pathway after treatment. This favorable response was concomitant with a noteworthy reduction of iron levels after the successful clinical intervention (Figs. 3D, E, s4E, F). Indeed, iron treatment significantly boosted the production of steroids, including cholesterol and lanosterol, in MM cells (Fig. s4G). The suppression of key genes governing cholesterol production, namely HMGCR, DHCR7, and DHCR24 (Fig. s4H–J), limited the role of iron in promoting cholesterol production (Fig. 3F, G). Similarly, knockdown of LSS gene (Fig. s4K), which catalyzes the last step of lanosterol production, interrupted the role of iron in boosting lanosterol production (Fig. 3H). These findings strongly indicate that iron enhances cholesterol and lanosterol production by activating sterol biosynthetic pathway.

Next, we optimized an in vitro co-culture system to screen DEGs in BMSCs interacting with MM.1S. In the co-cultured MM.1S cells, 158 genes were up-regulated, while 178 genes were downregulated (Fig. s5A). Significantly upregulated genes were found to be enriched in steroid pathway and cholesterol biosynthesis pathway, as indicated by GO and KEGG enrichment analysis (Fig. s5B, C). GSEA analysis further demonstrated that BMSCs promoted the activation of steroid biosynthesis (Fig. 3I). Subsequently, gas chromatography-mass spectrometry (GC–MS) was employed on control and co-cultured MM.1S cells, identifying 39 up-regulated and 19 down-regulated metabolites in MM.1S cells (Fig. 3J). Consistent with the RNA-seq results, KEGG analysis revealed that these upregulated metabolites were enriched in steroid biosynthesis, ferroptosis, glutathione metabolism, biosynthesis of unsaturated fatty acids, and pyrimidine metabolism (Fig. 3K). GSEA analysis also indentified that these pathways were positively correlated with coculture treatment (Fig. s5D–G). In the steroid biosynthesis pathway, cholesterol and lanosterol accumulation was observed (Fig. 3L). Notably, administration of the iron chelator, Deferoxamine (DFO), effectively mitigated the BMSCs-induced increase of cholesterol and lanosterol production (Fig. 3M). Furthermore, knockdown of key genes governing steroid biosynthesis, including HMGCR, DHCR7, DHCR24, and LSS, dramatically attenuated the stimulatory effect of BMSCs on cholesterol and lanosterol production (Fig. 3N, O). Collectively, these findings emphasize that BMSCs activate sterol biosynthetic pathway in MM cells through iron accumulation.

### Lanosterol enhances the sensitivity of MM cells to RSL3

**Fig. 4:**
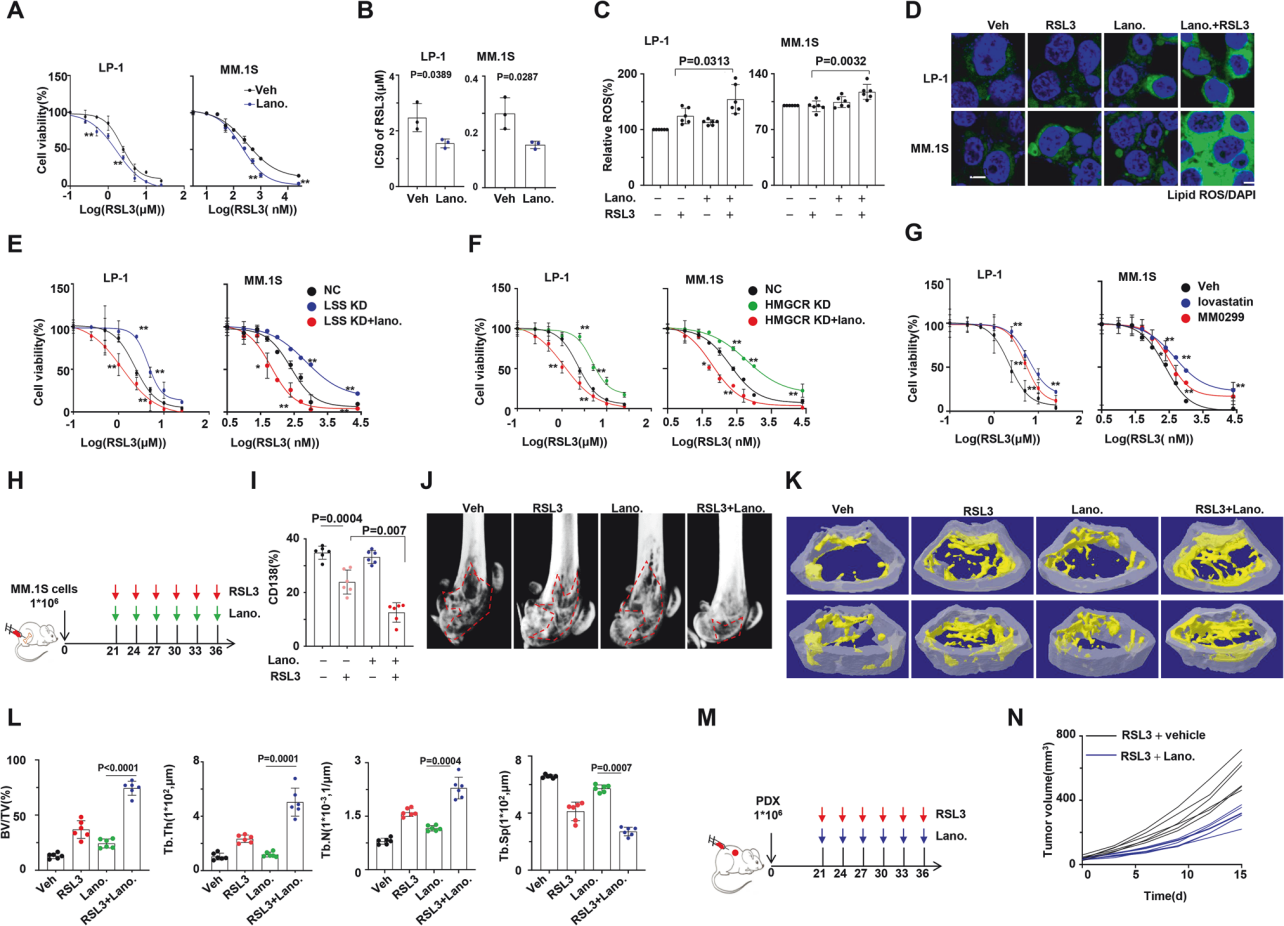
Lanosterol triggers ROS accumulation, thereby sensitizing MM cells to ferroptosis inducers via their interaction with BMSCs. **A** Alternation of IC50 to RSL3 in MM cells in the absence or presence of lanosterol (5 µM) supplementation, and **B** statistical analysis of IC50 (*n* = 3). **C** Lipid ROS levels of RSL3 treated MM cells in the absence or presence of lanosterol (5 µM) supplementation (*n* = 6). **D** Representative confocal images of MM cells loaded with oxidized formed C11-BODIPY (*n* = 3). **E** Alternation of IC50 to RSL3 in NC and LSS-KD MM cells in the absence or presence of lanosterol (5 µM) supplementation (*n* = 3). NC none target control. **F** The alternation of IC50 to RSL3 in NC and HMGCR-KD MM cells in the absence or presence of lanosterol (5 µM) supplementation (*n* = 3). NC, none target control. **G** Alternation of IC50 to RSL3 in MM cells in the absence or presence of lovastatin (10 nM) or MM0299 (2 nM) supplementation (*n* = 3). Lovastatin, selective inhibitor of HMGCR. MM0299, selective inhibitor of LSS. **H** Schematic diagram illustrating the treatment of intra-bone model derived from NSG mouse (*n* = 6). **I** Infiltration of CD138 positive cells in bone marrow of mouse femurs bearing MM.1S cells. **J**, **K** Representative micro-CT reconstructions and 3D reconstructions of bone trabecula in metaphyseal region of mouse femurs bearing MM.1 S cells. **L** Measurement of the percentage of bone volume to total volume (BV/TV), cortical thickness, number of bone trabecula, and trabecula separation in the metaphyseal region of the mice femur bearing MM.1S cells. **M** Schematic diagram illustrating the treatment of patient-derived model (PDX) using NSG mouse (*n* = 6). **N** Tumor growth in PDX model treated with RSL3 with or without lanosterol. Tumor volume = 1/2(L*W^2^) mm^3^, where L presents the length and W represents the width of the tumor. *P < 0.05. **P < 0.01. *P* values are determined by unpaired two-sided *t*-tests with Welch’s correction.

To explore the connection between ferroptosis and steroid pathway, we examined the impact of steroids supplementation on MM cell’s response to RSL3. Remarkably, lanosterol sensitized MM cells to RSL3 (Fig. 4A, B), and increased ROS accumulation in MM cells (Fig. 4C, D). However, cholesterol supplementation did not exhibit a similar effect as lanosterol (Fig. s6A–C). Suppression of key regulators of lanosterol synthesis, including LSS and HMGCR, by gene interference or using their inhibitors, significantly alleviated RSL3-induced cell death. This effect was substantially rescued by lanosterol administration (Fig. 4E–G). Using an intra-bone mouse model of MM cell growth (Fig. 4H), we confirmed that lanosterol prominently eliminated CD138^+^ plasma cells in bone marrow when combined with RSL3 (Fig. 4I). In addition, a significant remission in bone lesions was observed in the RSL3 treatment group, especially when lanosterol was supplemented (Fig. 4J). This was further validated by 3D reconstruction of the metaphyseal regions (Fig. 4K) and quantification of bone volume and microarchitecture (Fig. 4L). Moreover, lanosterol effectively re-sensitizes MM cells to RSL3 in the PDX model (Fig. 4M, N). These findings strongly indicate that lanosterol accumulation enhances the sensitivity of BMSCs interacting MM cells to RSL3.

### CD40-CD40L mediates BMSC-MM interaction

**Fig. 5:**
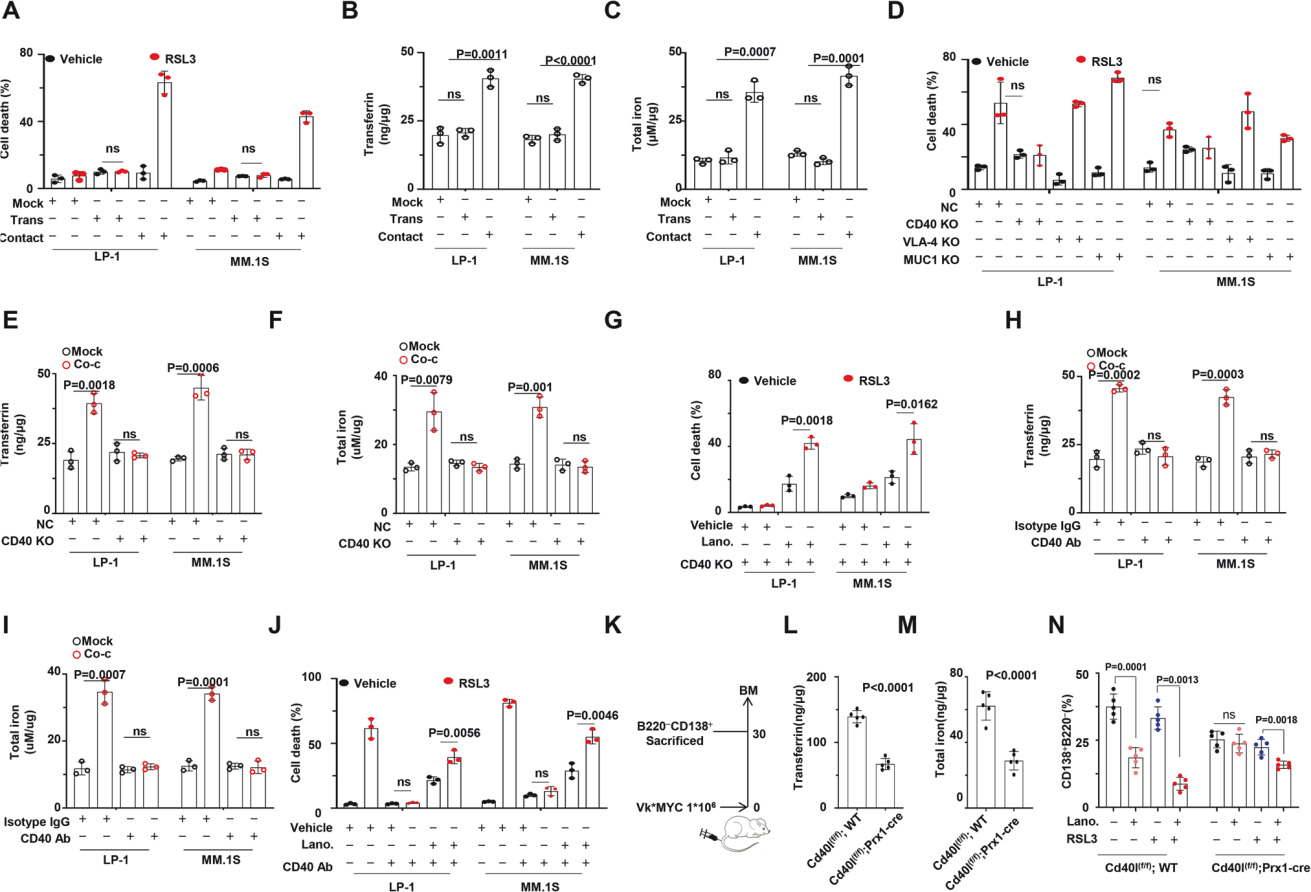
CD40/CD40L signaling mediates BMSCs and MM cells interaction. **A** Statistical analysis of ferroptosis in MM cells interacting with BMSCs by cell-cell contact or transwell model induced by RSL3 (*n* = 3). Mock represents MM cells only, Trans represents indirect contact between MM cells and BMSCs, and Contact represents direct contact between MM cells and BMSCs. **B** ELISA analysis of transferrin levels in MM cells interacting with BMSCs by cell–cell contact or transwell model (*n* = 3). Mock represents MM cells only, Trans represents indirect contact between MM cells and BMSCs and Contact represents direct contact between MM cells and BMSCs. **C** Total iron levels in MM cells interacting with BMSCs by cell-cell contact or transwell model (*n* = 3). Mock represents MM cells only, Trans represents indirect contact between MM cells and BMSCs, and Contact represents direct contact between MM cells and BMSCs. **D** Statistical analysis of ferroptosis of NC and target gene KO MM cells interacting with BMSCs (*n* = 3). NC, none target control. **E** ELISA analysis of transferrin levels in NC and CD40 KO MM cells interacting with BMSCs (*n* = 3). NC, none target control. **F** Total iron levels in NC and CD40 KO MM cells interacting with BMSCs (*n* = 3). NC, none target control. **G** Statistical analysis of ferroptosis of NC and CD40 KO MM cells interacting with BMSCs with or without lanosterol supplementation, lanosterol (10 μM), (*n* = 3). **H** ELISA analysis of transferrin levels in BMSCs interacting MM cells with or without anti-CD40 neutralizing antibody (*n* = 3). **I** Total iron levels in BMSCs interacting with MM cells with or without anti-CD40 neutralizing antibody (*n* = 3). **J** Statistical analysis of ferroptosis in BMSCs interacting with MM cells with or without anti-CD40 neutralizing antibody, in combination with or without lanosterol (10 μM), (*n* = 3). **K** Schematic diagram illustrating the treatment of Vk*MYC Vk12653 MM model (*n* = 5). **L** ELISA analysis of transferrin levels in CD138^+^B220^-^ cells of Cd40l^(f/f)^; WT and Cd40l^(f/f)^;Prx1-cre mouse-derived Vk*MYC Vk12653 MM model (*n* = 5). **M** Total iron levels in CD138^+^B220^-^ cells of Cd40l^(f/f)^; WT and Cd40l^(f/f)^;Prx1-cre mouse-derived Vk*MYC Vk12653 MM model (*n* = 5). **N** Statistical analysis of flow cytometry assay showing infiltration extent of CD138^+^B220^-^ cells of Cd40l^(f/f)^; WT and Cd40l^(f/f)^;Prx1-cre mouse-derived Vk*MYC Vk12653 MM model (*n* = 5). *P* values are determined by unpaired two-sided t-tests with Welch’s correction.

Given existing evidence that BMSCs interact with MM cells through both direct and indirect manners [23], we sought to elucidate how BMSCs facilitate the connection between MM cells and ferroptosis. In two in vitro co-culture systems, ferroptosis induced by RSL3 was only observed in the cell-cell contact model, not in the transwell system (Fig. 5A). Additionally, a remarkable augmentation of Transferrin and total iron was observed exclusively in the cell-cell contact condition (Fig. 5B, C). Next, we delved into identifying the specific cell-adhesion molecule responsible for mediating the interaction between BMSCs and MM cells. Drawing from the previous report [24], we selectively deleted three major molecules on MM cells, VLA-4, MUC1, and CD40, respectively (Fig. s7A–C). Intriguingly, only CD40 depletion attenuated RSL3-induced ferroptosis (Fig. 5D). This observation was supported by the fact that CD40 depletion on MM cells did not result in an upregulation of Transferrin or total iron levels in the cell-cell contact model (Fig. 5E, F). Notably, supplementation of lanosterol significantly restored the sensitivity to RSL3 in CD40-KO MM cells within the cell-cell contact system (Fig. 5G). Moreover, the use of anti-CD40 neutralizing antibody in the cell-cell contact system counteracted the BMSC-induced augmentation of Transferrin and total iron levels (Fig. 5H, I). Anti-CD40 neutralizing antibody abolished the cytotoxicity of RSL3 on MM cells in the co-cultured system, while lanosterol effectively rescued the cytotoxicity of RSL3 in the presence of anti-CD40 neutralizing antibody (Fig. 5J). Similarly, when CD40L was depleted in BMSCs, Transferrin and total iron levels in the co-cultured MM cells were barely augmented (Fig. s7D, E), and supplementation of lanosterol resensitized MM cells to RSL3 (Fig. s7F). To further investigate the role of the CD40/CD40L pathway in mediating BMSC-MM interaction, we established the Vk*MYC Vk12653 mouse model in the Cd40l^fl/fl^;Prx-Cre (CD40l-TKO) mice (Figs. 5K, s7G). The levels of Transferrin and total iron in CD138^+^B220^-^ cells derived from bone marrow of Cd40l-TKO mice were significantly suppressed compared to those in the Cd40l^fl/fl^;WT mice (Fig. 5L, M). Simultaneously, RSL3-triggered ferroptosis was attenuated in Cd40l-TKO mice, and the supplementation of lanosterol reinstated the sensitivity of MM cells to RSL3 (Fig. 5N). All these results strongly indicate that CD40-CD40L pathway serves as the the key bridge between BMSCs and MM cells, potentiating ferroptosis-based therapy in MM.

### Targeting GPX4 is CD40/CD40L dependent in vivo

**Fig. 6:**
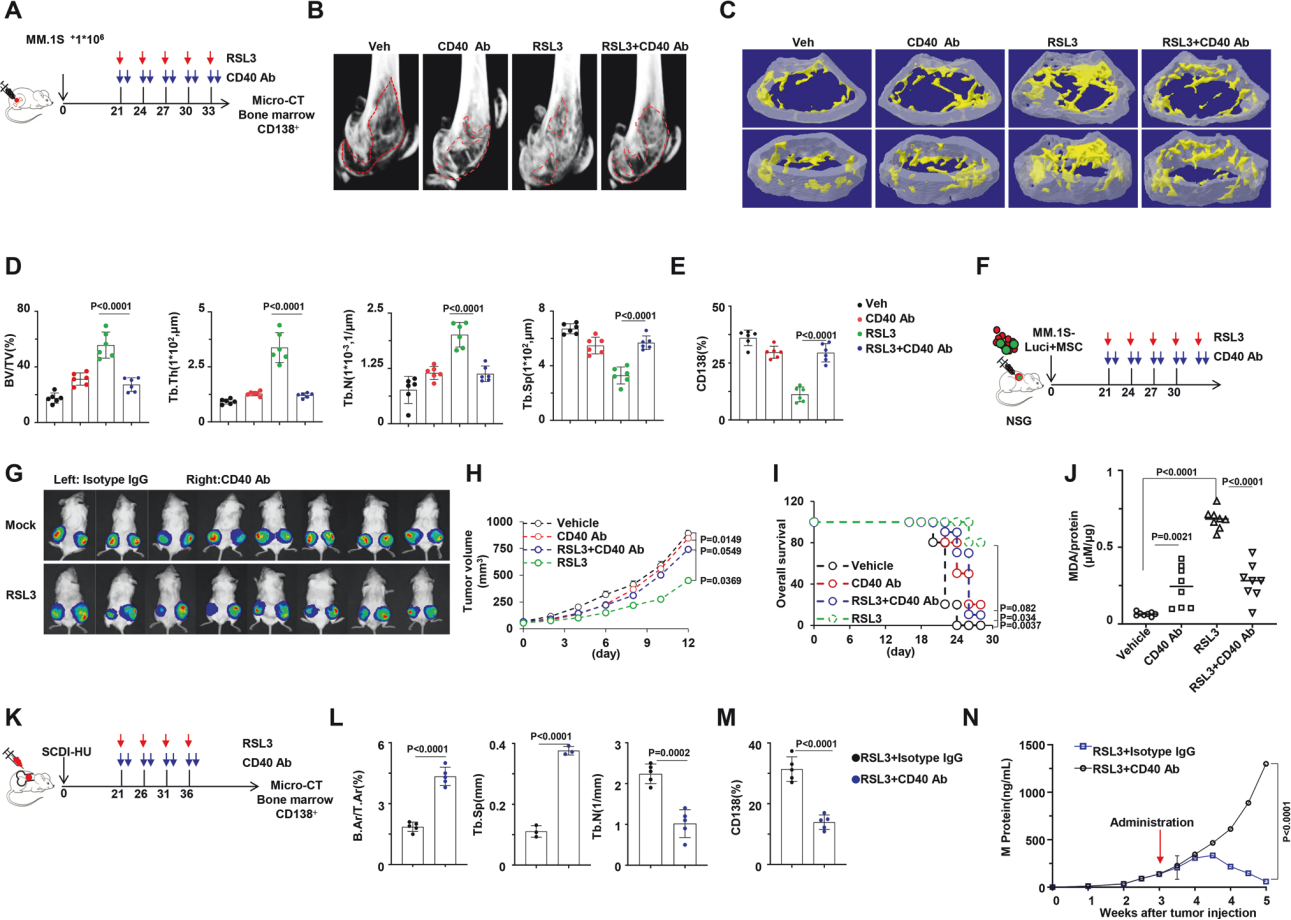
BMSC cells increase MM cell sensitivity to GPX4 inhibitor via CD40/CD40L-mediated direct interaction in vivo. **A** Schematic diagram illustrating the treatment of intra-bone model derived from NSG mice (*n* = 8). **B**, **C** Representative micro-CT reconstructions and 3D reconstructions of bone trabecula in metaphyseal region of mouse femurs bearing MM.1S cells. **D** Measurement of the percentage of bone volume to total volume (BV/TV), cortical thickness, number of bone trabecula, and trabecula separation in the metaphyseal region of the mice femur bearing MM.1S cells. **E** CD138 positive cells infiltration in bone marrow of mouse femurs bearing MM.1S cells. **F** Schematic diagram illustrating the treatment of xenograft model using NSG mice by inoculation of the mixture of MM.1S-luci and BMSCs (*n* = 8). **G** Tumor size of MM.1S-luci cells mixed with BMSC cells generated a xenograft model in NSG mice treated with RSL3 in combination with or without CD40 neutralizing antibody, left, Isotype IgG; Right, anti-CD40 neutralizing antibody. **H**, **I** Tumor growth and survival rate of each corresponding group. Tumor volume = 1/2(L*W^2^) mm, where L presents the length and W represents the width of tumor. Mice were sacrificed when the tumor achieved 15 mm. **J** MDA levels of tumor tissue from each corresponding group. **K** Schematic diagram illustrating the SCID-HU mouse model (*n* = 5). **L** Measurement of mean cortical area fraction (B.Ar/T.Ar), number of bone trabecula (Tb.N), and trabecula separation (Tb.Sp) of SCID-hu mouse model bearing patient-derived CD138^+^ plasma cells. **M** Showing CD138 positive cells infiltration of SCID-hu model bearing patient-derived CD138^+^ plasma cells. **N** Showing M protein production of SCID-hu model bearing patient-derived CD138^+^ plasma cells. Data present as mean ± s.e.m. *P* values are determined by unpaired two‐sided t‐tests with Welch’s correction.

To validate the role of CD40/CD40L in mediating the interaction between BMSCs and MM cells in vivo, we established the intra-bone MM models using NSG mice (Fig. 6A). Significant alleviation of the bone lesion was observed in the RSL3 treatment group; however, this effect was attenuated by the administration of anti-CD40 neutralizing antibody (Fig. 6B–D). Concurrently, anti-CD40 neutralizing antibody reversed the cell death of CD138^+^ plasma cells induced by RSL3 (Fig. 6E). Moreover, when luciferase-expressing MM.1 S cells, with or without BMSCs, were bilaterally inoculated into two flanks of NSG mice (Fig. 6F), RSL3 administration exhibited a remarkable anti-MM effect only in the presence of BMSCs. However, this effect was diminished when combined with anti-CD40 neutralizing antibody (Fig. 6G), as evidenced by measurements of tumor volume (Fig. 6H) and overall survival rate (Fig. 6I). Measurement of malondialdehyde (MDA) as a lipid peroxidation marker indicated that RSL3 administration triggered lipid oxidation production in tumor tissues, but this effect was remarkably abolished by treatment of anti-CD40 neutralizing antibody (Fig. 6J). Finally, the SCID-hu model was established to validate the role of CD40/CD40L in bridging human BMSCs and MM cells (Fig. 6K) [25]. Our results demonstrated that anti-CD40 neutralizing antibody, but not the isotype control, evidently abolished the anti-MM effect of RSL3, as indicated by heavier bone lesion (Fig. 6L), increased infiltration of CD138^+^ plasma cells in the transplanted fetal bone (Fig. 6M), and resulted in a higher level of M protein in mouse blood (Fig. 6N). Taken together, these results strongly suggest that targeting GPX4 in MM cells occurs through CD40/CD40L mediated interaction within bone marrow niche.

## Discussion

Bone marrow niche has been widely regarded as an important contributor to MM progression and treatment resistance, yet its fundamental role and intricate mechanism remain incompletely understood. In this study, we discover a previously unknown role of BMSCs in enhancing the sensitivity of MM cells to ferroptosis-based therapy. Mechanistically, the interaction with BMSCs through CD40/CD40L pathway upregulates Transferrin and iron levels in MM cells. Iron overload subsequently promotes the accumulation of lanosterol through the activation of steroid biosynthesis activation, thereby sensitizing MM cells to RSL3. Our findings provide a new perspective on understanding the bone marrow micromilieu’s impact on MM progression and also shed light on developing alternative approaches for managing refractory or relapsed MM patients who have shown resistance to PIs-based apoptosis regimens.

In this study, BMSCs were identified as key players in sensitizing MM cells to ferroptosis-based therapy, as evidenced by their ability to enhance MM cell sensitivity to RSL3 and alleviate MM-caused bone lesions. Previous researches have established that tumor environment can modulate the susceptibility of tumor cells to ferroptosis by promoting or suppressing ferroptosis through the action of immune cells or the release of specific metabolites from tumor environment [15–18]. Our study positions BMSCs, known for supporting the survival and chemo-resistance of MM cells, as potential contributors to a conducive environment for MM therapy. The consistent sensitivity to RSL3 between in vitro-induced BR and WT MM cells further emphasizes the indispensable role of BMSCs in this context. To date, limited studies have delved into the relationship between ferroptosis and hematological malignancies, especially MM. While traditional drugs like APR-246 and artesunate have demonstrated the induction of ferroptosis of acute myeloid leukemia cells and Burkitt lymphoma cells, respectively [26, 27], only one group explored the relationship between ferroptosis and MM cells, identifying FTY720 as a ferroptosis inducer to MM cells [28]. Zhang et al. found that system X_c_^−^ inhibitor erastin exhibited an anti-lymphoma effect [29]. However, our results showed that MM cells were sensitive to RSL3 but exhibited considerable tolerance to erastin. This difference may be attributed to the distinct types of tumor cells and low glutamate metabolism state in MM cells [30]. Thus, our study adds significant value to the existing research on ferroptosis in MM.

We observed elevated iron levels in BMSCs-interacting MM cells, and the iron, in turn, activated the steroid biosynthesis pathway, leading to the generation of lanosterol and cholesterol. Indeed, other components in bone marrow niche were reported to regulate iron level in MM cells, for example, Camiolo et al. reported that macrophages could drive bortezomib resistance of MM cells by elevating iron level [31], which also supported our hypothesis. The alteration of metabolism caused by tumor environment has been reported to influence the susceptibility of cancer cells to ferroptosis. For instance, lactate has been associated with resistance to RSL3 in solid tumor cells [17], while n-3 and n-6 polyunsaturated fatty acids have been linked to increased sensitivity to RSL3 in cancer cells [18]. A recent study also reported that 7-DHC, followed by cholesterol and Desmo, could mediate the ferroptosis suppression in liver cells [32]. However, cholesterol accumulation did not change the sensitivity to RSL3 in MM cells. Lanosterol, another product of steroid biosynthesis pathway, was found to sensitize MM cells to RSL3 by generating ROS in our study. This aligns with the findings of another group that reported lanosterol’s ability to induce ROS in macrophage [33]. Our study not only enhances the understanding of the role of steroid biosynthesis pathway in ferroptosis but also implies that metabolites may exhibit diverse roles between solid and hematological cancers.

Furthermore, we observed higher iron levels in CD138+ samples obtained from bone marrow aspiration of non-response patients and a more aggressive Vk*MYC Vk12653 MM model, suggesting that relapsed and refractory MM patients may be the better candidates for ferroptosis-based-therapy in the clinic. While BMSCs have been implicated in promoting chemo-resistance by increasing the iron content in MM cells, our in vitro induction of BR MM cells did not render them more sensitive to RSL3, emphasizing the BMSCs dependence in ferroptosis-based-therapy in MM cells. Given that BMSCs can promote chemoresistance of MM cells through both direct (cell-cell contact) and indirect (cytokines) ways, we further delved into deciphering which pathway mediates the interaction between BMSCs and MM cells. Among known adhesion molecules, including VCAM1/VLA-4, CD40/CD40L, and MUC1/ICAM1 [12], we identified that CD40/CD40L signaling acts as the bridge between BMSCs and MM cells, sensitizing MM cells to ferroptosis-based-therapy by promoting iron accumulation. In summary, our study has brought new strategies for MM treatment, especially for relapsed and refractory MM patients, suggesting that besides inducing apoptosis, ferroptosis could also be a key target for MM therapy.

## Conclusion

Our current study discovered a previously unrecognized role of BMSCs in sensitizing MM cells to ferroptosis-based therapy by generating ROS from lanosterol through the activation steroid biosynthesis pathway. The clinical merit of our study lies in providing a rationale for using GPX4 inhibitor RSL3 to develop non-apoptotic strategies for managing refractory or relapsed MM patients who have shown resistance to PIs.

## Materials and methods

### Patient samples collection and patient-derived xenograft (PDX) model

Diagnosis of relapsed/refractory (RR), response and none-response in MM patients was conducted based on the criteria established by the International Myeloma Working Group (IMWG), the Revised International Staging System (R-ISS) for MM, and the key exclusion criteria [34]. Blood serum and CD138^+^ cells from both MM patients and healthy controls were detailed in the previous study [34]. For PDX model, unsorted BM mononuclear cells from RRMM patients after a bortezomib-based treatment regimen were intratibially implanted into randomized grouping NOD.Cg-PrkdcscidIl2rgtm1Wjl/SzJ mice (femal, 4 weeks old, *n* = 6). After the indicated duration, BTZ alone or in combination with lanosterol was administered twice a week for 2 weeks, (BTZ intraperitoneal injection, 5 mg/kg; cholesterol intra-tumor injection, 20 μg/mice), and xenograft model mice were sacrificed upon reaching a tumor length of 15 mm. Double-blind trials were applied in the PDX model.

### Vk*MYC Vk12653 transplant mouse models of MM

The Vk*MYC Vk12653 transplant mouse model was established according to Dr. Bergsagel PL’s protocol [35]. Briefly, 1 × 10^6^ harvested cells were injected via tail vein into 5 weeks old C57BL/6 WT, Cd40l^(f/f)^; WT, and Cd40l^fl/fl^;Prx1^Cre/+^ mice, respectively. Cell pellets of peripheral blood, spleen, and bone marrow were obtained from recipient mice for 3 weeks, 4 weeks, and 6 weeks along with serum from peripheral blood. The obtained cells and serum were used for subsequent experiments. Double-blind trials were applied in the Vk*MYC Vk12653 transplant mouse model.

### Statistical analysis

Data are shown as mean ± SD for a minimum of three independent experiments. Group differences were determined using paired two-sided Student’s t-test or two-way ANOVA. A *P*-value less than 0.05 was considered statistically significant compared to the controls.

More materials and methods could be found in the supplementary methods file.

### Supplementary information

Supplementary Figures

Supplementary methods

Supplementary Resources

## Data Availability

All necessary data to evaluate the conclusions presented in the paper are provided in the paper and/or the Supplementary Materials. The RNA-seq data are accessible at the Gene Expression Omnibus database under accession number GSE214768. Requests for any materials related to this study should be directed to Zhiqiang Liu.
