# Supplementary material for: Bone marrow stromal cells dictate lanosterol biosynthesis and ferroptosis of multiple myeloma
Source: Oncogene. 2024 Apr 9;43(21):1644–53. doi: 10.1038/s41388-024-03020-5 (PMC11108777; doi:10.1038/s41388-024-03020-5)
Supplement: Supplementary file 1 — Supplementary Figures [file 41388_2024_3020_MOESM1_ESM.docx]

**Supplementary Figures and Figure Legends**

**
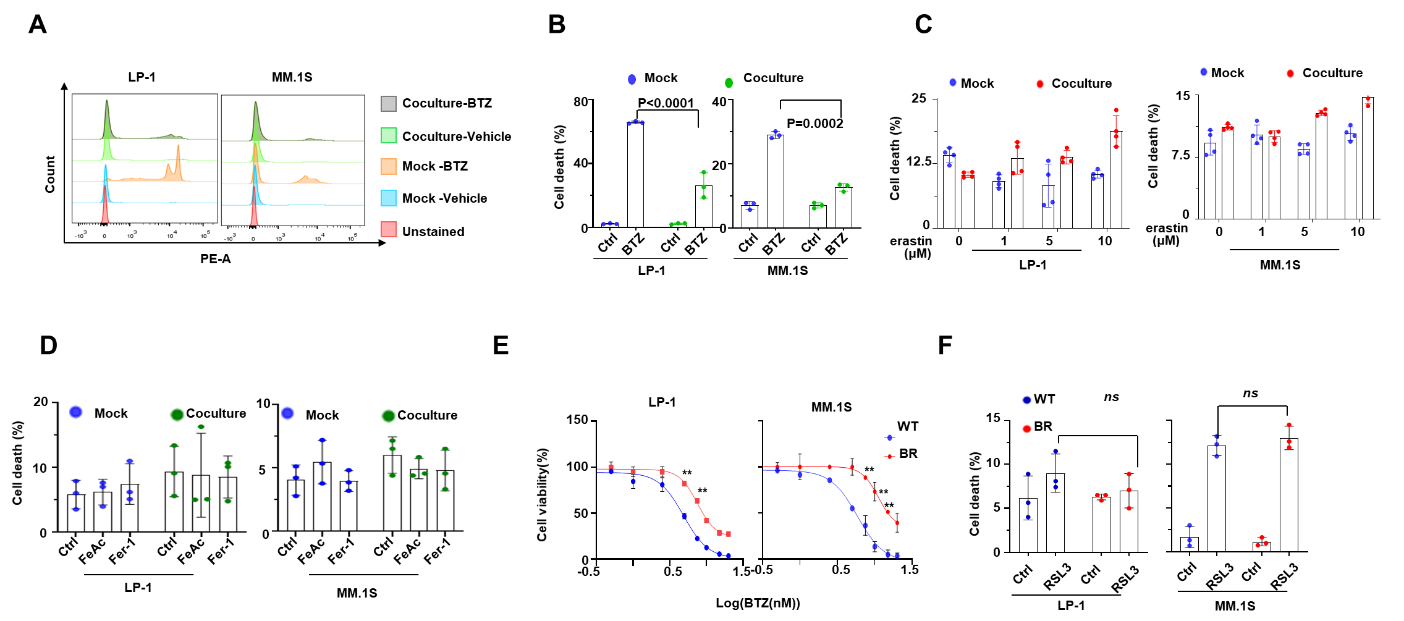
**

**S Figure 1**. **BMSC-interacting MM cells are more resistant to BTZ treatment but tolerant to erastin.**

(**A**) Representative flow cytometry assay showing dead cells determined by PI in mock and co-cultured MM cells induced by BTZ for 48 hours, and (**B**) statistical analysis of flow cytometry results (n=3). Mock indicates MM cells only, and Co-culture indicates MM cells co-cultured with BMSCs. (**C**) Statistical analysis of flow cytometry assay showing cell death determined by PI in mock and co-cultured MM cells induced by erastin, a System xc^−^ inhibitor (n=4). Mock indicates MM cells only, and Co-culture indicates MM cells co-cultured with BMSCs. (**D**) Statistical analysis of flow cytometry assay showing cell death determined by PI in mock and co-cultured MM cells induced by FeAc (300μM) and Fer-1 (100μM) (n=3). Mock indicates MM cells only, and Co-culture indicates MM cells co-cultured with BMSCs. (**E**) The alternation of IC50 to BTZ in WT and BR cells (n=3). (**F**) Statistical analysis of RSL3-induced cell death in WT and BR MM Cells, identified by flow cytometry assay with PI (n=3). **, P<0.01. P values are determined by unpaired two‐sided t‐tests with Welch's correction.


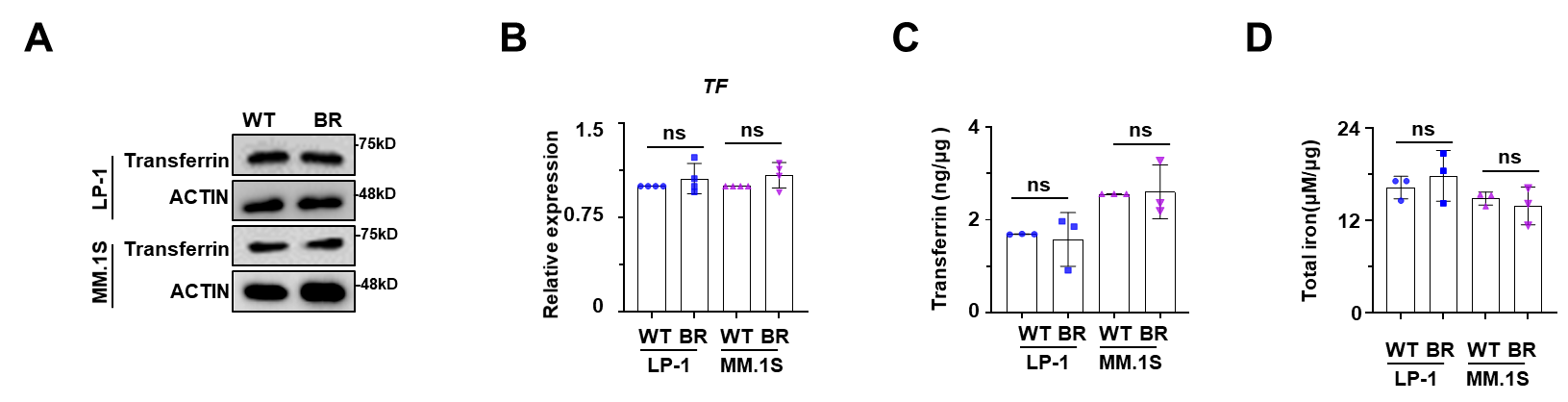


**S Figure 2**. **No difference in iron metabolism was identified between in vitro-induced BR MM cells and WT MM cells.**

(**A**) Representative western blotting showing transferrin levels in WT and BR MM cells (n=3). (**B**) Real-time PCR results showing mRNA expression of *TF* in WT and BR MM cells (n=3). (**C**) ELISA analysis of transferrin levels in WT and BR MM cells (n=3). (**D**) Total iron levels in WT and BR MM cells (n=3). P values are determined by unpaired two‐sided t‐tests with Welch's correction.

**
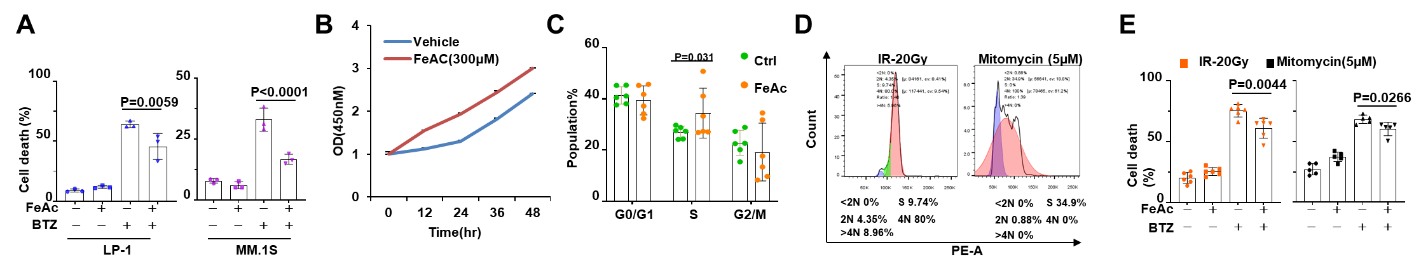
**

**S Figure 3**. **Iron promotes BR in MM cells.**

(**A**) Representative flow cytometry assay showing dead cells determined by PI in control and co-cultured MM cells induced by BTZ (7.5nM) and FeAc (300μM) for 48 hours (n=3). (**B**) Cell viability assay showing proliferation rate of MM cells treated with FeAc. (**C**) Cell cycle analysis of Vehicle- and FeAc-treated MM cells (n=6). (**D**) Representative flow cytometry assay showing cell cycle of LP-1 cells in the presence of mitomycin (5μM) or ionizing radiation (IR) (20Gy) for 48 hours (n=5). (**E**) Representative flow cytometry assay showing dead cells determined by PI in control and co-cultured LP-1 cells induced by BTZ (7.5nM) and FeAc (300μM) in the presence of mitomycin (5μM) or IR (20Gy) for 48 hours (n=5). P values are determined by unpaired two‐sided t‐tests with Welch's correction.


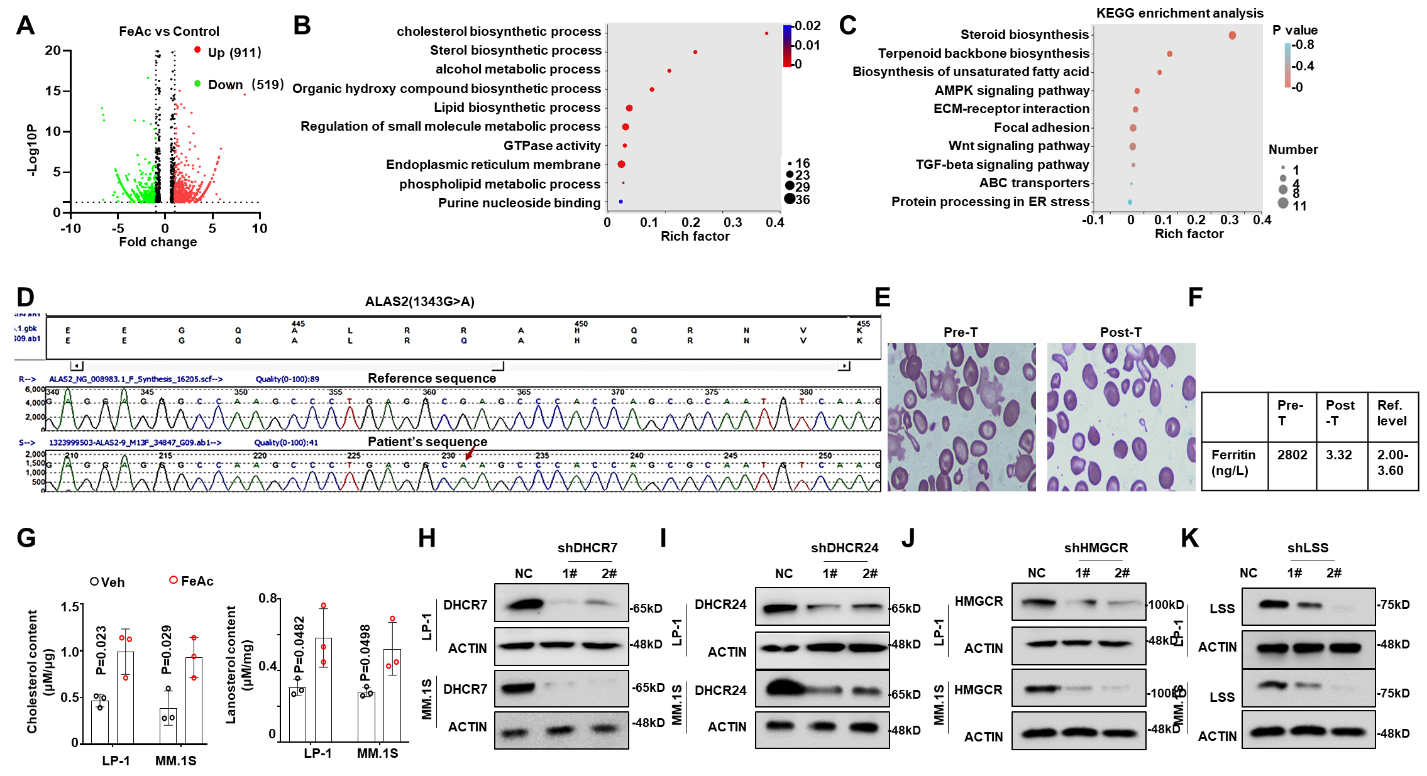


**S Figure 4**. **Iron accumulation activates cholesterol biosynthesis pathway in MM cells.**

(**A**) Volcano plot showing differentially expressed genes in FeAc (300µM)-treated MM.1S cells. The number of genes downregulated or upregulated in MM.1S cells is shown on the top (fold change > 1.5; adjusted p-value < 0.05). (**B**) Gene Ontology (GO) analysis showing the top 20 biological processes in FeAc (300µM)-treated MM.1S cells. (**C**) KEGG analysis showing the top 10 pathways in FeAc (300µM)-treated MM.1S cells. (**D**) Mutation site of ALAS2 in samples from the iron-overload patient. (**E**) Mutation blood smear of bone marrow samples from the iron-overload patient before (pre-T) or after treatment (post-T). (**F**) Ferritin levels in samples from the iron-overload patient before (pre-T) or after treatment (post-T). (**G**) Cholesterol and lanosterol content in MM cells with or without FeAc (300µM) supplementation (n=3). (**H-K**) Representative western blotting quantifying the protein knockdown of DHCR7, DGCR24, HMGCR, and LSS in MM cells (n=3). NC, none target control. P values are determined by unpaired two‐sided t‐tests with Welch's correction.


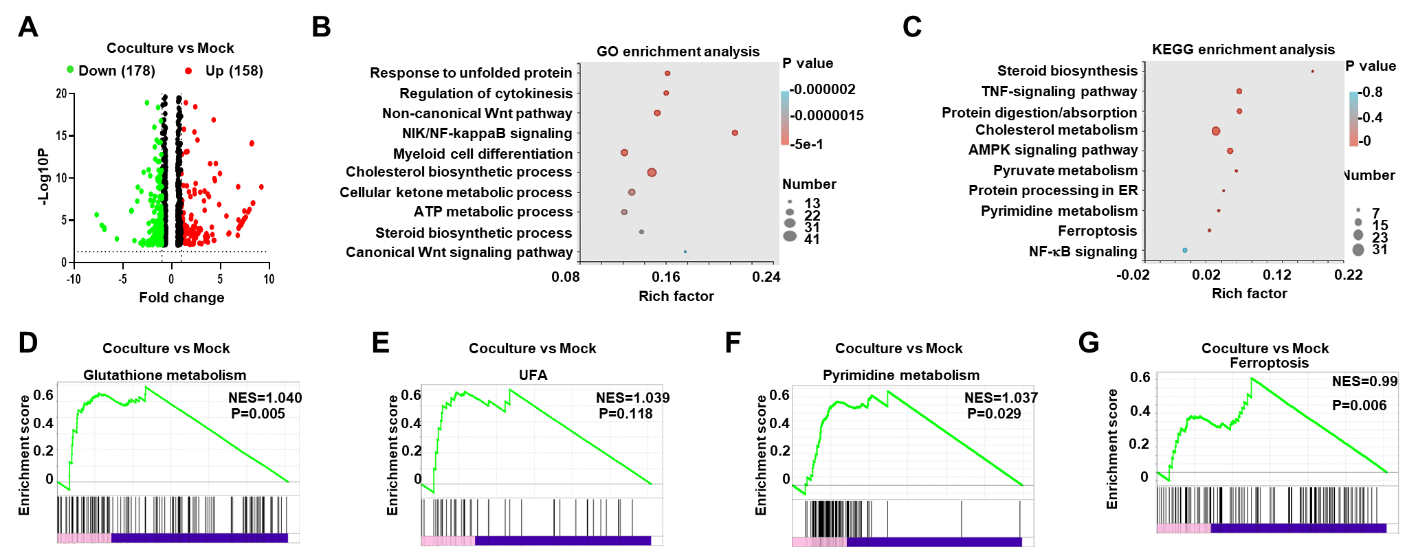


**S Figure 5**. **Interaction with BMSCs activates ferroptosis pathway in MM cells.**

(**A**) Volcano plot showing differentially expressed genes in co-cultured MM cells. The number of genes downregulated or upregulated in MM cells is shown on the top (fold change > 1.5; adjusted p-value < 0.05). Mock indicates MM.1S cells only, and Co-culture indicates MM.1S co-cultured with BMSCs. (**B**) Gene Ontology (GO) analysis showing the top 10 biological processes enriched by upregulated genes in co-cultured MM cells. (**C**) KEGG analysis showing the top 10 pathways enriched by upregulated genes in co-cultured MM cells. (**D**) GSEA analysis showing gene enrichment in glutathione metabolism pathway in mock and co-cultured MM.1S cells. (**E**) GSEA analysis showing gene enrichment in unsaturated fatty acid biosynthesis pathway in mock and co-cultured MM.1S cells. (**F**) GSEA analysis showing gene enrichment in pyrimidine metabolism pathway in mock and co-cultured MM.1S cells. (**G**) GSEA analysis showing gene enrichment in ferroptosis pathway in mock and co-cultured MM.1S cells. P values are determined by unpaired two‐sided t‐tests with Welch's correction.


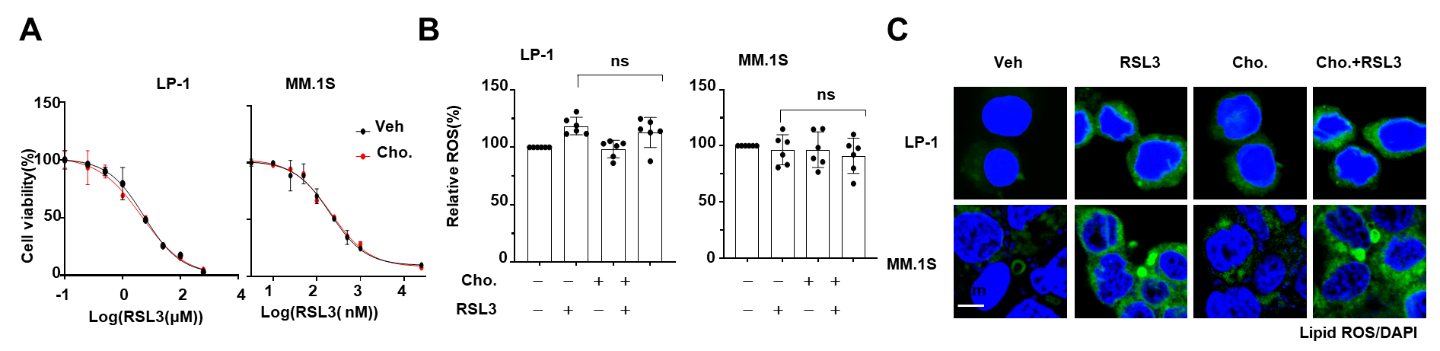


**S Figure 6**. **Cholesterol accumulation in co-cultured MM cells fails to trigger ROS accumulation.**

(**A**) The alternation of IC50 to RSL3 in MM cells in the absence or presence of cholesterol supplementation (n=3). (**B**) Lipid ROS levels of RSL3-treated MM cells with or without cholesterol supplementation (n=6). (**C**) Representative confocal images of MM cells loaded by oxidized formed C11-BODIPY (n=3). P values are determined by unpaired two‐sided t‐tests with Welch's correction.

**
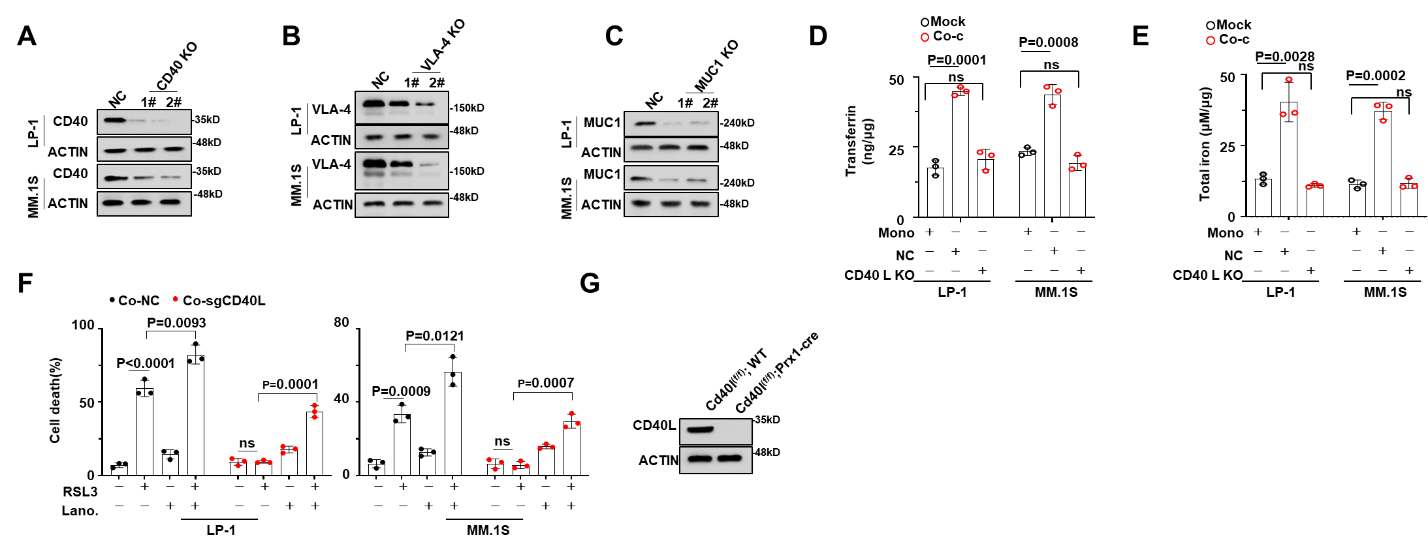
**

**S Figure 7**. **CD40/CD40L mediates the interaction between BMSCs and MM cells.**

(**A-C**) Representative western blotting assessing the knockout of CD40, VLA-4, and MUC1 in MM cells (n=3). NC, none target control. (**D**) ELISA analysis of transferrin levels in MM cells interacted with NC BMSCs and CD4OL-KO BMSCs (n=3). NC, none target control. (**E**) Total iron levels in MM cells interacted with NC BMSCs and CD4OL-KO BMSCs (n=3). NC, none target control. (**F**) Statistical analysis of ferroptosis content in MM cells interacted with NC BMSCs and CD4OL-KO BMSCs (n=3). NC, none target control. (**G**) Representative western blotting demonstrating the knockout of CD40L in BMSC cells derived from Cd40l^(f/f)^; WT and Cd40l^(f/f)^;Prx^cre/+^ mice (n=3). P values are determined by unpaired two‐sided t‐tests with Welch's correction.
