# Supplementary material for: Bone marrow stromal cells dictate lanosterol biosynthesis and ferroptosis of multiple myeloma
Source: Oncogene. 2024 Apr 9;43(21):1644–53. doi: 10.1038/s41388-024-03020-5 (PMC11108777; doi:10.1038/s41388-024-03020-5)
Supplement: Supplementary file 2 — Supplementary methods [file 41388_2024_3020_MOESM2_ESM.docx]

**Supplementary Methods**

**Resources and cultures of cells**

Resources and cultures of HEK-293T and MM cells and induction of BR cells have been introduced in our previous report^1^. Authentications of HEK-293T and MM cells were identified using short tandem repeat (STR) (Biowing Biotech, Shanghai, China). Primary BMSCs were collected from femurs of bone marrow samples. Diluted 5mL bone marrow samples were added on the top of Ficoll-Paque in a sterile 15mL centrifuge tube, followed by centrifuging at the speed of 800g for 20 minutes, and then mononuclear cells in the middle of the tube were collected and rinsed twice; at last these cells were resuspended into a 10cm dish. The media was replaced every 3 days. BMSCs were passaged to 2 parts when the fusion degree reached at 80-90% and the passages 3 could be used for the subsequent experiments. MM cells were cocultured with BMSCs at the ratio of 5:1 for 24 hours, and then were separated by differential adhesion for further experiments. Mycoplasma-free was assured using Universal Mycoplasma Detection Kit (ATCC, Manassas, VA, USA).

**Real-time PCR and western blotting**

Real-time PCR and western blotting were performed as before^1^. Primers of the real-time PCR for target genes were listed in the **supplementary resources,** and the fold change of expression was calculated using the formula of 2^-ΔΔCt^. For Western blotting, cells lysis was quantified and electrophoresed by SDS-PAGE gel system. After incubation with corresponding primary antibodies as listed in the **supplementary resources** and secondary antibodies sequentially, target proteins were visualized by a chemiluminescence system (Millipore, Los Angeles, CA USA).

**Elisa**

Transferrin levels were measured according to the manufacturer’s instruction of a Human Transferrin ELISA Kit and Mouse Transferrin ELISA Kit, respectively. Briefly, the corresponding samples were added into the plate with primary antibody for incubation at 37°C for 90 min. Biotinylated detection antibody working solution was added for incubation at 37°C for 60 min. HRP conjugate working solution was added for incubation at 37°C for 30 min. Substrate Reagent was added for incubation in dark at 37°C for 15min. At last stop solution was added and optical density was determined at 450 nm.

**Iron detection**

Total iron detection was measured according to the manufacturer’s instruction of Iron Assay kit. Briefly, samples were added into a 96-well plate followed with 5µL of Iron Reducer to each well. Samples were mixed and were incubated at 37°C for 30 minutes. 100µL Iron Probe was added to each well. The plate was incubated at 37°C for 60 minutes in the dark and determined at OD 593 nm.

**Cell viability detection**

1*10^4^ LP-1 cells or 3*10^4^ MM.1S were cultured in each well of 96-well plates, and then treated with RSLL3 or BTZ or RSL3 for 48 hr in the absence or presence of cholesterol(40μg/Ml), lanosterol(10μM), uridine(100μM), orotate(100μM) and dihydroorotate(100μM). Then, samples were read at 490 nm by a Microplate Reader 550 (Bio-Rad Laboratories, Richmond, CA, USA) to calculate cell viability (%) using the formula = treatment group (OD value)/control group (OD value) ×100.

**Flow cytometry analysis**

For cell death identification, the preprocessing MM cells were collected and stained with propidium iodide on the ice for 15 minutes. For cell cycle detection, the preprocessing MM cells were collected and stained according to the manufacture’s instruction of Cell Cycle and Apoptosis Analysis Kit. For CD138 positive cells infiltration and GPX4 level of CD138 positive cells derived from constructed mouse model, bone marrow contents, peripheral blood and spleen of constructed mouse model were flushed into 1.5mL tubes and 10 percent cell pellets were stained with PE-CD138 antibody at 4℃ in the dark for 30 minutes. Samples were fixated and permeablized by Fixation/Permeablization Kit. After being stained with anti-GPX4 and corresponding secondary antibody, the samples were detected by flow cytometry and analyzed with FlowJo X software (BD Biosciences, New Jersey, USA).

**RNA-sequencing**

Cell pellets were obtained as designed, total RNA was extracted from two biological replicates. RNA quality was assessed using a 2100 Expert Bioanalyzer (Agilent) and sent for library preparation and sequencing using the Illumina Hiseq2000 platform of Majorbio Biotech (Shanghai, China). The data were analyzed on the free online Majorbio I-Sanger Cloud Platform UENCING ([www.i-sanger.com](http://www.i-sanger.com)).

**Ester extraction and Cholesterol quantification**

At least 3*10^6^ indicated cells were lysed by 80% ethanol, followed by 3 times of freeze-thawed cycles and 3 times of ultrasonic disruptor with 40% power. After being centrifuged at speed of 14000g for 10 minutes, deposit was performed for protein concentration quantification by bicinchoninic acid (BCA) and supernatant was transferred to another centrifuge tube containing 1mL methyl tert-butyl ether (MTBE) for 1 hour vortex. 0.2mL tri-distilled water was added and left to set for 10 minutes in the fume cupboard. Ester dissolved supernatant was transferred to another new centrifuge tube and dried by nitrogen in the fume cupboard. 100% methanol dissolved extraction, which was filtrated by the 0.22μm filter, was analyzed by the cholesterol detection kit and quantified by protein concentration.

**Gas Chromatograph Mass Spectrometer**

­Untarget metabonomics sequencing and analysis with untarget database of GC-MS from Lumingbio of over 1*10^7^ cocultured MM cells were performed by Oebiotech Co., Ltd. (Shanghai, China).

**Reference**

1 Jiang, H. *et al.* Posttranslational modification of Aurora A-NSD2 loop contributes to drug resistance in t(4;14) multiple myeloma. *Clin Transl Med* **12**, e744, doi:10.1002/ctm2.744 (2022).
