# Supplementary material for: Bone marrow stromal cells dictate lanosterol biosynthesis and ferroptosis of multiple myeloma
Source: Oncogene. 2024 Apr 9;43(21):1644–53. doi: 10.1038/s41388-024-03020-5 (PMC11108777; doi:10.1038/s41388-024-03020-5)
Supplement: Supplementary file 3 — Supplementary Resources [file 41388_2024_3020_MOESM3_ESM.docx]

**Key resources of this study**

| **Category** | **Source** | **Cat. No.** |
| --- | --- | --- |
| **Antibodies** | | |
| Anti-Transferrin Rabbit mAb | ABclonal Technology | A19130 |
| Anti-rabbit IgG | Proteintech | 30000-0-AP |
| Anti-rabbit β-actin | ABclonal Technology | AC006 |
| PE anti-mouse CD138 Antibody | Biolegend | 142504 |
| Anti-Glutathione Peroxidase 4 | Abcam | ab125066 |
| Goat Anti-Rabbit IgG H&L (Alexa Fluor® 488) | Abcam | ab150077 |
| APC anti-mouse/human CD45R/B220 | Biolegend | 103212 |
| Anti-rat HA-Tag (C29F4) | Cell signaling technology | #3724 |
| Anti-PARP | Cell signaling technology | 9532 |
| DHCR7 Rabbit pAb | ABclonal Technology | A8049 |
| HMGCR Rabbit pAb | ABclonal Technology | A1633 |
| DHCR24/Seladin-1 (C59D8) | Cell signaling technology | #2033 |
| FTH1 (D1D4) Rabbit mAb | Cell signaling technology | #4393 |
| ANTI-FLAG® M2-Peroxidase | Sigma-Aldrich | A8592 |
| HA-Tag (C29F4) Rabbit mAb | Cell signaling technology | #3724 |
| CD40 (E2Z7J) Rabbit mAb | Cell signaling technology | #86165 |
| Integrin α4 Antibody | Cell signaling technology | #4600 |
| MUC1 (D9O8K) XP® Rabbit mAb | Cell signaling technology | #14161 |
| **Chemicals, Peptides and Recombinant Proteins** | | |
| 3FLAG peptide | Sigma-Aldrich | F4799 |
| FLAG Peptide | Sigma-Aldrich | F3290 |
| Protein A/G magnetic | YEASEN | 36417ES |
| Pierce Protein G plus Agrose | Sigma-Aldrich | #22852 |
| ANTI-FLAG® M2 Affinity Gel | Sigma-Aldrich | A2220 |
| Pierce Glutathaione Agrose | Sigma-Aldrich | 16100 |
| InVivoMAb anti-human CD40 | Bxcell | BE0189 |
| Bortezomib (PS-341) | SelleckChem | S1013 |
| RSL3 | SelleckChem | S8155 |
| Ferric ammonium citrate | Sigma-Aldrich | 1185-57-5 |
| Deferoxamine | Sigma-Aldrich | Y0001934 |
| desferrioxamine | Sigma-Aldrich | 138-14-7 |
| Cholesterol | Sigma-Aldrich | 57-88-5 |
| Lanosterol | Sigma-Aldrich | 79-63-0 |
| Uridine | Sigma-Aldrich | 58-96-8 |
| Orotate | Sigma-Aldrich | 65-86-1 |
| Dihydroorotate | Sigma-Aldrich | 5988-19-2 |
| Mitomycin C | SelleckChem | S8146 |
| Puromycin 2HCL | SelleckChem | S7417 |
| C11 Bodipy 581/591 | Thermo Fisher | D3861 |
| Calicium-AM | sigma | 148504-34-1 |
| deferiprone | sigma | 30652-11-0 |
| Ferric Citrate | sigma | 1185-57-5 |
| IVISbrite D-Luciferin Potassium Salt Bioluminescent Substrate | PerkinElmer | 122799 |
| Propidium iodide | Solarbio | 25535-16-4 |
| Erastin | SelleckChem | S7242 |
| Mitotracker | Beyotime | C1049B |
| MG132 | SelleckChem | S2619 |
| **Enzymes** | | |
| RNase A, DNase and protease-free | Thermo Fisher | EN0531 |
| Proteinase K Solution, ChIP grade | Thermo Fisher | 26160 |
| Benzonase Nuclease | Sigma-Aldrich | E1014-25KU |
| FastAP Thermosensitive Alkaline Phosphatase | Thermo Fisher | EF0651 |
| BsmBI | NewEngland Biolabs | R0580S |
| NotI-HF | NewEngland Biolabs | R3189S |
| BamHI | NewEngland Biolabs | R0136S |
| XbaI | NewEngland Biolabs | R0145S |
| EcoRI | NewEngland Biolabs | R0101S |
| KpnI-HF | NewEngland Biolabs | R3142S |
| HindIII | NewEngland Biolabs | R104S |
| XhoI | NewEngland Biolabs | R0146S |
| CutSmart Buffer | NewEngland Biolabs | 137204S |
| NEBuffer1 | NewEngland Biolabs | B7001S |
| NEBuffer2 | NewEngland Biolabs | B7002S |
| NEBuffer3 | NewEngland Biolabs | B7003O |
| NEBuffer4 | NewEngland Biolabs | B7004S |
| T4 DNA Ligase | NewEngland Biolabs | M0202S |
| 10×Buffer for T4 DNA ligase | NewEngland Biolabs | B0202S |
| **Plasmids** | | |
| lentiCRISPRv2 puro | Addgene | |
| pLKO.1 | Addgene | |
| DHCR7 sh#1 Custom clone | Self-construction | |
| DHCR7 sh#2 Custom clone | Self-construction | |
| DHCR24 sh#1 Custom clone | Self-construction | |
| DHCR24 sh#2 Custom clone | Self-construction | |
| HMGCR sh#1 Custom clone | Self-construction | |
| HMGCR sh#2 Custom clone | Self-construction | |
| GV364-GPX4-N-3xFlag | genechem | |
| pITA-insert | Gift from Dr. Chen, Tianjin Medical University | |
| PSPAX_2_ | Gift from Dr. Xudong Wu, Tianjin Medical University, Dept. Cell Biology | |
| PMD_2_G | Gift from Dr. Xudong Wu, Tianjin Medical University, Dept. Cell Biology | |
| pITA-insert- GPX4-N-3xFlag | Self-construction | |
| pITA-insert-GPX4-c-HA | Self-construction | |
| SUMO1-HA | Addgene | |
| SUMO2-HA | Addgene | |
| SUMO3-HA | Addgene | |
| lentiCRISPRv2 puro | Addgene | |
| CD40 CRISPR1# Custom clone | Self-construction | |
| CD40 CRISPR2# Custom clone | Self-construction | |
| VLA-4 CRISPR1# Custom clone | Self-construction | |
| VLA-4 CRISPR2# Custom clone | Self-construction | |
| MUC1 CRISPR1# Custom clone | Self-construction | |
| MUC1 CRISPR2# Custom clone | Self-construction | |
| CD40L CRISPR1# Custom clone | Self-construction | |
| CD40L CRISPR2# Custom clone | Self-construction | |
| **Critical Commercial Assays** | | |
| Human TF(Transferrin) ELISA Kit | Elabscience | E-EL-H6028 |
| Mouse TF (Transferrin) ELISA Kit | Elabscience | E-EL-M1184 |
| Iron Assay Kit (Colorimetric) | Abcam | ab83366 |
| Ficoll-Paque^TM^ PLUS | cytiva | 17144002 |
| Pierce™ Silver Stain Kit | Thermo fisher Scientific | 24612 |
| Cell Cycle and Apoptosis Analysis Kit | Beyotime | C1052 |
| Fixation/Permeablization Kit | BD Bioscience | 554714 |
| Transwell(0.4μm) | Corning Incorporated Costar | 3450 |
| EvaGreen 2X qPCR MasterMix | ABI | MasterMix-R |
| 5×All-In-One RT MasterMix | abm | G490 |
| Pierce BCA Protein Assay Kit | Thermo SCIENTIFIC | 23225 |
| Cell Counting Kit-8 | APEXBIO | K1018 |
| AxyPrep Plasmid Miniprep Kit | AXYGEN | 183 AP-MN-P-250G |
| Plasmid Maxi Kit(25) | QIAGEN | 12163 |
| EnVision G12 Doublestain System,Rabbit/Mouse(DAB+/Permanent Red) | Dako | K5361 |
| NuPAGE 4-12% Bis-Tris Gel | Invitrogen | NP0335BOX |
| Phosphatase Inhibitor Cocktail(100×) | Cell Signaling | 587OS |
| LS Columns(25 columns) | Miltenyi Biotec | 130-042-401 |
| TRIzol Reagent | Ambion, Life Science | 15596018 |
| Opti-MEM®I(1×) Reduced Serum | Gibco, Life Technologies | 31985-070 |
| Polyethylenimine | Polyscience | 23699-1 |
| Hexadimethrine bromide | Sigma-Aldrich | 107689 |
| 1Kb DNA Ladder | Genstar | M114 |
| 100bp DNA ladder | Genstar | M016 |
| Primers sequence | | |
| hGAPDH-F | TTGCCCTCAACGACCACTTT | |
| hGAPDH-R | TGGTCCAGGGGTCTTACTCC | |
| DHCR24-sh#1-F | CCGGGCTGAATAGCATTGGCAATTACTCGAGTAATTGCCAATGCTATTCAGCTTTTTG | |
| DHCR24-sh#1-R | AATTCAAAAAGCTGAATAGCATTGGCAATTACTCGAGTAATTGCCAATGCTATTCAGC | |
| DHCR24-sh#2-F | CCGGGCATTGGCAATTACTACAAGCCTCGAGGCTTGTAGTAATTGCCAATGCTTTTTG | |
| DHCR24-sh#2-R | AATTCAAAAAGCATTGGCAATTACTACAAGCCTCGAGGCTTGTAGTAATTGCCAATGC | |
| DHCR24-sh#3-F | CCGGGCCGTGGTTCTTTAAGCATGTCTCGAGACATGCTTAAAGAACCACGGCTTTTTG | |
| DHCR24-sh#3-R | AATTCAAAAAGCCGTGGTTCTTTAAGCATGTCTCGAGACATGCTTAAAGAACCACGGC | |
| DHCR7-sh#1-F | CCGGGCAACCCAACATTCCCAAAGCCTCGAGGCTTTGGGAATGTTGGGTTGCTTTTTG | |
| DHCR7-sh#1-R | AATTCAAAAAGCAACCCAACATTCCCAAAGCCTCGAGGCTTTGGGAATGTTGGGTTGC | |
| DHCR7-sh#2-F | CCGGGCGCCAGAGACTGCAAATTCACTCGAGTGAATTTGCAGTCTCTGGCGCTTTTTG | |
| DHCR7-sh#2-R | AATTCAAAAAGCGCCAGAGACTGCAAATTCACTCGAGTGAATTTGCAGTCTCTGGCGC | |
| DHCR7-sh#3-F | CCGGGGAAGTGGTTTGACTTCAAGCCTCGAGGCTTGAAGTCAAACCACTTCCTTTTTG | |
| DHCR7-sh#3-R | AATTCAAAAAGGAAGTGGTTTGACTTCAAGCCTCGAGGCTTGAAGTCAAACCACTTCC | |
| HMGCR-sh#1-F | CCGGGCATGTCAGTTCTTGCCAACTCTCGAGAGTTGGCAAGAACTGACATGCTTTTTG | |
| HMGCR-sh#1-R | AATTCAAAAAGCATGTCAGTTCTTGCCAACTCTCGAGAGTTGGCAAGAACTGACATGC | |
| HMGCR-sh#2-F | CCGGGCTTGTGTGTCCTTGGTATTACTCGAGTAATACCAAGGACACACAAGCTTTTTG | |
| HMGCR-sh#2-R | AATTCAAAAAGCTTGTGTGTCCTTGGTATTACTCGAGTAATACCAAGGACACACAAGC | |
| HMGCR-sh#3-F | CCGGGCAGATACTTCTAAGGTTTCACTCGAGTGAAACCTTAGAAGTATCTGCTTTTTG | |
| HMGCR-sh#3-R | AATTCAAAAAGCAGATACTTCTAAGGTTTCACTCGAGTGAAACCTTAGAAGTATCTGC | |
| hHMGCS1-1F | TATTCCAAGCCCTGCCAAGA | |
| hHMGCS1-1R | GACAGCTGCTTCAGGTTCTG | |
| hHMGCS1-2F | AGCTGCTGTCTTCAATGCTG | |
| hHMGCS1-2R | CTGCTCCAACTCCACCTGTA | |
| hHMGCR-1F | AAAGCCTGGCTCGAAACATC | |
| hHMGCR-1R | CGTGCAAATCTGCTAGTGCT | |
| hHMGCR-2F | GGTGGGACCAACCTACTACC | |
| hHMGCR-2R | TTGCATGCTCCTTGAACACC | |
| hMVD-1F | TATGCCTGCCTAGCCTACAC | |
| hMVD-1R | GCCACTTCTGAGAGGTCACT | |
| hMVD-2F | GCGATGAAGAGCTGGTTCTG | |
| hMVD-2R | TAACTGGTCCTGGTGCAGAG | |
| hFDFT1-1F | TCTGGAAGACCAGCAAGGAG | |
| hFDFT1-1R | AGTTCATTCAGGCACTGCAC | |
| hFDFT1-2F | CCCAACGATCTCCCTTGAGT | |
| hFDFT1-2R | AAACTCTGCCATCCCAATGC | |
| hSC5D-1F | TCTTCTGTGCAACACTGAGC | |
| hSC5D-1R | TCCAGCAAGAACAGTGCAAC | |
| hSC5D-2F | GGATCTTGTACTCCGTGTTGC | |
| hSC5D-2R | GCTCAGTGTTGCACAGAAGA | |
| hCYP51A-1F | TGGATGGAGGTTTCAGCCAT | |
| hCYP51A-1R | AGACTGTCTGCGTTTCTGGA | |
| hCYP51A-2F | GGTCTACCTGATCCGTCTGG | |
| hCYP51A-2R | GCATGCCCAAGGAATGGAAT | |
| hMSMO1-1F | GGATAAGCCAGAGACATGGGA | |
| hMSMO1-1R | GCACAACCAAAGCATCTTGC | |
| hMSMO1-2F | ATGCACATCCTTTGGAGACT | |
| hMSMO1-2R | ATGCCGAGAACCAGCATAGA | |
| hEBP-1F | GAGGGCTGGTTCGTTCTCTA | |
| hEBP-1R | TGTCACCCAGGATGTATCGG | |
| hEBP-2F | AGTATGCCAAGGGAGACAGC | |
| hEBP-2R | AGCTGTGATGGTTTCCATGC | |
| hLSS-1F | ACCCTGTTCCCAGAGATGTG | |
| hLSS-1R | GGCGAAGTCCTCCACATAGA | |
| hLSS-2F | TGTATGAGCACCACCACAGT | |
| hLSS-2R | TGGAGACATGCTCCTGGAAG | |
| hSQLE-1F | AAGGTCTTGATGCCCAGGTT | |
| hSQLE-1R | AACTTTGCATTGGGCTCTGC | |
| hSQLE-2F | TCTGCCACAGATGATTCCCT | |
| hSQLE-2R | TACAGAAAGCAGCCCAACAG | |
| hDHCR7-1F | TTCTCGCCCACCATCATCTT | |
| hDHCR7-1R | CGAGGGTTAAACTCGATGCC | |
| hDHCR7-2F | CGCCAGAGACTGCAAATTCA | |
| hDHCR7-2R | CGCCCATTGAAGAACAGCTT | |
| hDHCR24-1F | TTCTATGCCGTACCCTGGTC | |
| hDHCR24-1R | GAAACGCAGCTTGACGTACT | |
| hDHCR24-2F | ATGAGGCAGCTGGAGAAGTT | |
| hDHCR24-2R | TTCTCTCGCAGCTTGTGGTA | |
| hTF-1F | GCTGTTGCTGTGGTGAAGAA | |
| hTF-1R | GCCACTGCTTTCTCAAGAGG | |
| hTF-2F | GCAGCACCTATTTGGAAGCA | |
| hTF-2R | GGAGTGATGAGGTGGAGCAT | |
| MUC1-oligo-1 | CACCGCTACGATCGGTACTGCTAGGGGG | |
| MUC1-oligo-1-1 | AAACCCCCCTAGCAGTACCGATCGTAGC | |
| MUC1-oligo-2 | CACCGGGCTACGATCGGTACTGCTAGG | |
| MUC1-oligo-2-1 | AAACCCTAGCAGTACCGATCGTAGCCC | |
| MUC1-oligo-3 | CACCGGGCTACGATCGGTACTGCTAGGG | |
| MUC1-oligo-3-1 | AAACCCCTAGCAGTACCGATCGTAGCCC | |
| CD40LG-oligo-1 | CACCGGATTCTCTCGAATCTACCGGGGG | |
| CD40LG-oligo-1-1 | AAACCCCCCGGTAGATTCGAGAGAATCC | |
| CD40LG-oligo-2 | CACCGAGATTCTCTCGAATCTACCGGGG | |
| CD40LG-oligo-2-1 | AAACCCCCGGTAGATTCGAGAGAATCTC | |
| CD40LG-oligo-3 | CACCGCGAATCTACCGGGGGACTTTAGG | |
| CD40LG-oligo-3-1 | AAACCCTAAAGTCCCCCGGTAGATTCGC | |
| ITGA4-oligo-1 | CACCGAGTAGCCGAACAGCGTGTTGTGG | |
| ITGA4-oligo-1-1 | AAACCCACAACACGCTGTTCGGCTACTC | |
| ITGA4-oligo-2 | CACCGCAACACGCTGTTCGGCTACTCGG | |
| ITGA4-oligo-2-1 | AAACCCGAGTAGCCGAACAGCGTGTTGC | |
| ITGA4-oligo-3 | CACCGGAGCTGTTCGCACGTCTGGCCGG | |
| ITGA4-oligo-3-1 | AAACCCGGCCAGACGTGCGAACAGCTCC | |
| CD40-oligo-1 | CACCGAGGAATTCGCTTTCACCGCAAGG | |
| CD40-oligo-1-1 | AAACCCTTGCGGTGAAAGCGAATTCCTC | |
| CD40-oligo-2 | CACCGATTCGCTTTCACCGCAAGGAAGG | |
| CD40-oligo-2-1 | AAACCCTTCCTTGCGGTGAAAGCGAATC | |
| CD40-oligo-3 | CACCGACCCCAGTGCGTGCGCTGTTGGG | |
| CD40-oligo-3-1 | AAACCCCAACAGCGCACGCACTGGGGTC | |
